# Extending BigSMILES to non-covalent bonds in supramolecular polymer assemblies†

**DOI:** 10.1039/d2sc02257e

**Published:** 2022-09-15

**Authors:** Weizhong Zou, Alexis Martell Monterroza, Yunxin Yao, S. Cem Millik, Morgan M. Cencer, Nathan J. Rebello, Haley K. Beech, Melody A. Morris, Tzyy-Shyang Lin, Cleotilde S. Castano, Julia A. Kalow, Stephen L. Craig, Alshakim Nelson, Jeffrey S. Moore, Bradley D. Olsen

**Affiliations:** Department of Chemical Engineering, Massachusetts Institute of Technology Cambridge MA 02142 USA bdolsen@mit.edu +617 715-4548; Department of Chemistry, Northwestern University Evanston IL 60208 USA; Department of Chemistry, Duke University Durham NC 27708 USA; Department of Chemistry, University of Washington Seattle WA 98195 USA; Department of Chemistry, University of Illinois at Urbana-Champaign Urbana IL 61801 USA; Beckman Institute for Advanced Science and Technology, University of Illinois at Urbana-Champaign Urbana IL 61801 USA; Roxbury Community College Boston MA 02120 USA

## Abstract

As a machine-recognizable representation of polymer connectivity, BigSMILES line notation extends SMILES from deterministic to stochastic structures. The same framework that allows BigSMILES to accommodate stochastic covalent connectivity can be extended to non-covalent bonds, enhancing its value for polymers, supramolecular materials, and colloidal chemistry. Non-covalent bonds are captured through the inclusion of annotations to pseudo atoms serving as complementary binding pairs, minimal key/value pairs to elaborate other relevant attributes, and indexes to specify the pairing among potential donors and acceptors or bond delocalization. Incorporating these annotations into BigSMILES line notation enables the representation of four common classes of non-covalent bonds in polymer science: electrostatic interactions, hydrogen bonding, metal–ligand complexation, and π–π stacking. The principal advantage of non-covalent BigSMILES is the ability to accommodate a broad variety of non-covalent chemistry with a simple user-orientated, semi-flexible annotation formalism. This goal is achieved by encoding a universal but non-exhaustive representation of non-covalent or stochastic bonding patterns through syntax for (de)protonated and delocalized state of bonding as well as nested bonds for correlated bonding and multi-component mixture. By allowing user-defined descriptors in the annotation expression, further applications in data-driven research can be envisioned to represent chemical structures in many other fields, including polymer nanocomposite and surface chemistry.

## Abstract 2

Non-covalent BigSMILES enables the representation of donor/acceptor interactions and delocalized bonds for polymer assemblies.

## Graphical abstract

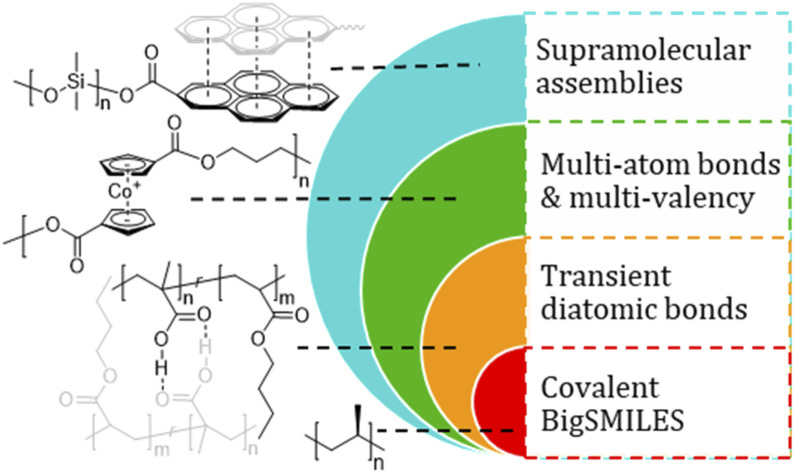

## 1 Introduction

Over the past decade, artificial intelligence and data-driven models have exhibited great potential in chemistry.^1–5^ These techniques have been readily adopted for many applications, such as synthetic data mining and digitalization,^6,7^ material property predictions,^8,9^ reaction pathway visualization,^10,11^ and molecular design,^12,13^ where a variety of chemical information must be transformed into machine-recognizable representations. For small molecules, most representations usually fall into one of the following classes: line notation,^14–16^ graph grammars,^17–19^ and 3D geometrical representations.^20–22^ Compared to the other representation methods, line notations offer a superior combination of human-readability and machine programing; consequently, line notation is the most widely used method for denoting chemical formulae.^6,12,23,24^

Among established line notations such as IUPAC nomenclature,^25^ InChI (International Chemical Identifier)^26^ and PubChem,^27^ the flexibility and versatility of SMILES (simplified molecular input line entry system) has made it ubiquitous in many data sorting and storage applications.^28–30^ However, SMILES notation is not without limitations: for instance, both multivalency and aromaticity cannot be properly annotated in SMILES syntax. To extend the use of SMILES strings, many derivatives including CurlySMILES,^31^ OpenSMILES,^32^ and more recently InChIfied SMILES^33^ have been developed. These efforts primarily focus on improving the syntax for small molecules with few^19,28,30^ being applicable to polymers and supra-molecules. Since a polymer representation corresponds to an ensemble of molecules with different chemical structures, any machine-recognizable syntax must fully capture this stochastic nature with minimal sacrifice to readability.

Recently, BigSMILES line notation was developed to capture the stochastic nature of polymer structure in a manner fully compatible with SMILES syntax. The utility of BigSMILES has been demonstrated in several database projects.^34–37^ In addition, to convert between BigSMILES strings and conventional chemical structure drawings, a parser, canonicalization schemes, and graphic editor programs have been developed to support its broader use.^38^ By adding stochastic bonding descriptors to the original syntax of SMILES, BigSMILES allows for iterative generation of molecular fragments of varying sizes and can readily be used to automatically extract chemical subgraphs and attach molecular fingerprints.^39^ A recent application of BigSMILES for the development of a polymer data schema, PolyDAT,^40^ provides a framework for storing data on the synthesis and chemical characterization of polymers needed for complete chemical description.

Expanding BigSMILES to accurately capture non-covalent bonds, which are critical to material structure in biomaterials, supramolecular, and colloidal chemistries, will enhance its potential in materials informatics.^41–44^ The stochastic nature of BigSMILES is inherently compatible with variable bonding patterns, providing an opportunity to extend BigSMILES to accommodate non-covalent bonds. In this work, a general annotation syntax for non-covalent bonds is introduced into the BigSMILES grammar, enabling the depiction of simple transient bonds with representative examples for coulombic interactions^45–47^ as well as hydrogen bonding.^48–52^ To build towards multi-atom bonds and multivalent interactions, such as metal–ligand coordination,^53,54^ π–π stacking,^55–58^ and host–guest complexation,^55,59^ an additional atom indexing feature is applied to specify the range of interactions. The capability of the above formalism is demonstrated by its use to encode a range of different supramolecular assemblies. By extending BigSMILES notation with these grammatical elements, important functionalities associated with non-covalent chemistry can be captured, substantially expanding the power of BigSMILES to serve as chemical identifiers in machine learning and material informatics.

## 2 Syntax

### 2.1 Overview on non-covalent BigSMILES

**Fig. 1:**
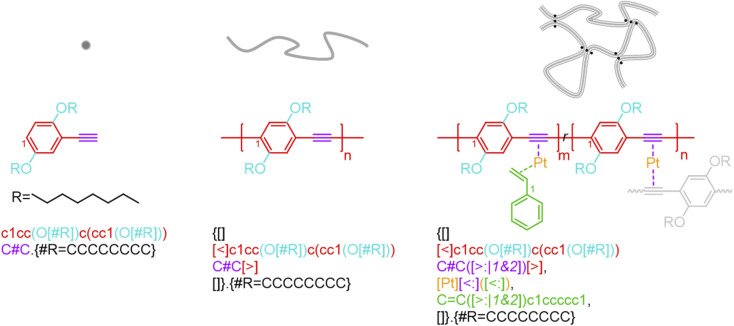
Illustration on the change of chemical structure, annotation syntax, and corresponding molecular topology by from left to right SMILES, BigSMILES, and non-covalent BigSMILES to present the network of dialkoxy-*p*-phenyleneethynylene derivative (EHO-OPPE) and Pt(styrene)_3_.^60^ Note that in the above figure, different parts of chemical structures are coded with the same colors as their representing strings. “[]” is used next to the curly brackets since end groups and the associated connectivity pattern to repeating units (RUs) are unspecified for the examples shown here.

BigSMILES includes descriptors of bonds and their associated indexing to depict molecular connectivity of various types, expressed systematically through an annotation formalism. As illustrated in Fig. 1, an integral element of BigSMILES notation is the use of stochastic bond descriptors enclosed in square brackets, *i.e.*, [>], [<] or [$] to represent the connectivity among different fragments of polymer molecules.^39^ [$] is to denote symmetric bonding, while the pair of [<] and [>] are for asymmetric bonds. Molecular fragments, or repeat units, are encoded under the syntax of SMILES, leaving bond descriptors as pseudo-atoms that represent the stochastic connections formed during polymerization. Non-covalent BigSMILES extends from the above framework to accommodate a broad variety of non-covalent chemistry enabling the representation of complementary interactions among potential donors and acceptors as well as delocalized bonds. This is achieved by encoding bond descriptors with a general donor–acceptor principle that captures the electronic nature of non-covalent interactions. It is further complemented by syntax, such as indexes and key/value pairs, whose presence in the bond annotation closely resembles the role of adjectives in a natural language. Fig. 1 exemplifies the use of these features to specify correlated bonding patterns and multi-component binding as well as to elaborate relevant attributes of non-covalent bonds including but not limited to coordination and number of delocalized electrons. Given users are also allowed to formulate their own context-annotated features, *i.e.*, key/value pairs, non-covalent BigSMILES offers a highly generalizable approach to represent diverse non-covalent chemistries and polymeric systems with foreseeable extensions to other less-common supramolecular interactions. Note that in what follows, for ease of reading, different parts of chemical structures and their representing segments of strings are coded with the identical colors.

### 2.2 General syntax for non-covalent bonds

As exemplified in Fig. 1, a new bond annotation scheme was developed with the intention to capture the electronic nature of a non-covalent bond. Motivated by both the rich annotation types and the customized encoding format in CurlySMILES,^31^ a general syntax for non-covalent bond annotation is given below:[X:|indices, key = value, …]

In the above notation, the non-covalent annotation is contained within a pair of square brackets and starts with a predefined bond descriptor “X” and colon “:”. The choices for X are “$”, “<”, or “>”, followed by atom indices for delocalization (*i.e.*, |*i* ∼ *j*) and a comma-delimited list of key/value pairs (*i.e.*, key1 = value1, key2 = value2 in Table 1) which serve to denote the type and other intrinsic attributes of the non-covalent bond. As in BigSMILES, positive integers can also be appended to the bond descriptor as the corresponding index. The choice of <:, >: or $: as the symbol for the bond depends upon the corresponding directionality of the electrons involved in a non-covalent bond. For atoms or ions that have lowest unoccupied molecular orbitals (LUMOs) that are able to accept electrons, otherwise known as electron acceptors, “A[<: …]” shall be used to represent electrons bonding towards the acceptor atom A. In contrast, “B[>: …]” indicates B is an atom with a highest occupied MO (HOMO) that is able to donate electrons, otherwise known as an electron donor, with bonding electrons being provided from donor atom B. The syntax [$: …] is then used for interactions that do not possess strong donor–acceptor character nor permanent dipole moment (for instance, π–π stacking). The coordination geometry (in metal–ligand complexations) and the number of electrons involved (in electrostatic interaction) can also be described through this syntax. Many other important attributes that are not necessarily required for the expression of a polymer chemical structure, such as physical state, surface morphology, molecular weight, and monomer composition, are preferentially stored in an associated data structure as they are fundamentally tied to characterization data. One such structure is the recently proposed PolyDAT.^40^ This philosophy of reducing the number of annotations also makes the properties in the data structure more searchable with traditional text-based queries,^5,6,23,24^ and machine recognizable BigSMILES strings may be used as the associated chemical identifiers.

**Table 1:** Predefined syntactic features for annotation of non-covalent bonds

Feature	Notation	Description
Bond symbol	<:, >:, $:	<: (>:) for atoms possess strong electron acceptor (donor) character or corresponding permanent dipole moment, otherwise $:
Bond index	Single integer	Indices for specification of pair-wise non-covalent interaction, *e.g.*, [<:1] and [>:1], [$:1] and [$:1], in Fig. 4d, 5a, 6
Delocalization	*x* ∼ *y*, *m*&*n*, !*z*	Indices for atoms involved in delocalization (Fig. 3), inclusive boundaries “*x*”, “*y*” for the range of indices with step of 1; indices of individual atom characters to include are combined by “&”; Exclamation symbol “!” is equivalent to logical operator “not”
Key/value	*cg* = …,	Coordination geometry defined by polyhedral symbol and coordination number,^61^*e.g.*, TP3 for trigonal planar-3 coordinates, in Fig. 5b
	*ne* = …	Number of electrons involved in the bonding, in Fig. 3a

**Fig. 2:**
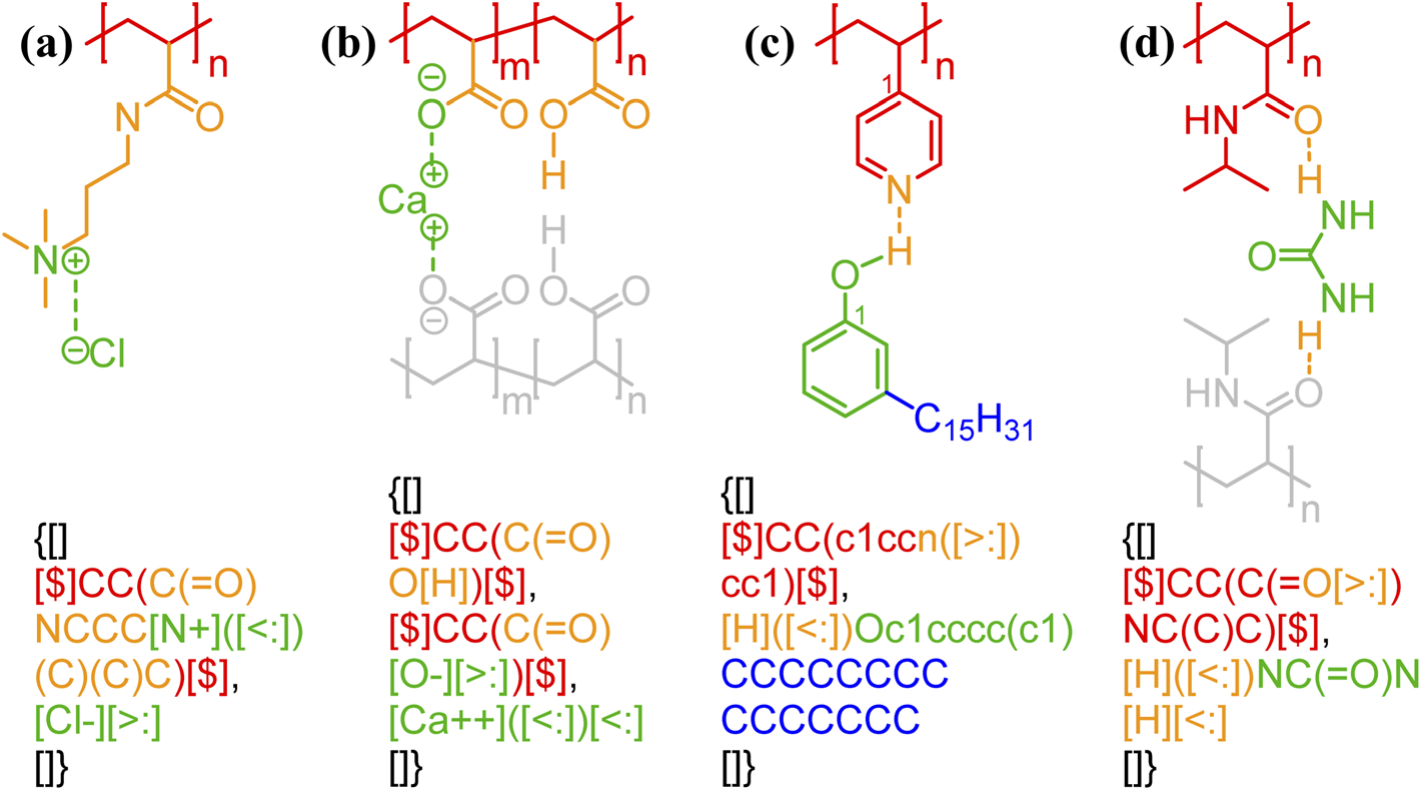
Non-covalent bond annotation in typical polyelectrolytes and hydrogen bonding polymers. Examples include (a) cationic poly(acrylamido-*N*-propyltrimethylammonium chloride) (polyAPTAC);^46^ (b) polyacrylic acid crosslinked by divalent calcium ions;^47^ (c) poly(4-vinylpyridine) (P4VP) hydrogen bonding with an alkylphenol;^48^ and (d) poly(*N*-isopropylacrylamide) (PNIPAM) crosslinked by urea.^51^ Note that for panel b, both neutral and deprotonated states of carboxylic group are indicated in the annotation expression. In all the above figure, different parts of chemical structures are coded with the same colors as their non-covalent BigSMILES strings.

One of the simplest implementations of the above scheme is for electrostatic bonds. In addition to representing a major class of polymeric materials in biological systems, polyelectrolytes are at the center of many industrial applications.^45–47^ Electrostatic bonds always occur between positively charged and negatively charged moieties, denoted with the bond descriptors <: (for electron acceptor, *i.e.*, cations) and >: (for electron donor, *i.e.*, anions), respectively. Two examples of these bonds are shown in Fig. 2a and b.

As another major category of non-covalent chemistry, hydrogen bonds have directionality and are therefore naturally captured with the notation <: and >: for lone pair electron acceptors and donors, respectively. Fig. 2c and d illustrate the basic syntax for annotating hydrogen bonding with more examples shown in Fig. S3–S5 in the ESI.† In Fig. 2c, alkylphenol molecules are hydrogen-bonded to poly(4-vinylpyridine) and need to be viewed similar as stochastic grafts (and thus included in the curly brackets) instead of deterministic end-groups. Hydrogen bonding also serves as one of the most common mechanisms for reversible bonding,^48–52^ and small-molecule hydrogen bond donors or acceptors can be added to trigger the formation of physical gels. The annotation of such gels is exemplified by the urea-crosslinked poly-(*N*-isopropylacrylamide) (PNIPAM) gels in Fig. 2d. Non-covalent BigSMILES is devised to contain single polymer connectivity (*via* bonds) among RUs and associated small molecules, as illustrated in Fig. 2. For complicated multi-component systems, such as coacervate complexes and hydrogels, the overall system should be represented as a combination of non-covalent BigSMILES strings with bonding interactions between molecules, whose detailed representation can be found in Fig. S6 in the ESI.†

### 2.3 Bond delocalization and atom indexing

**Fig. 3:**
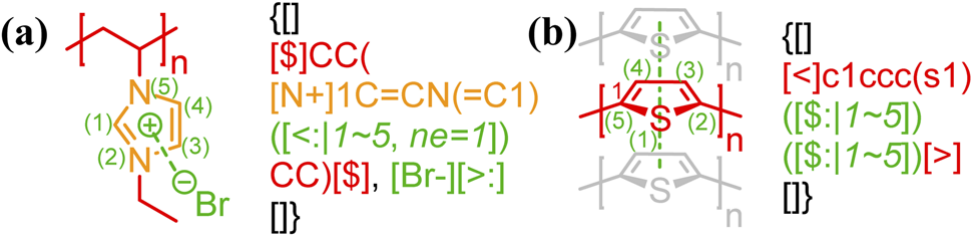
Annotation of delocalized bonds contributed by a group of atoms for (a) electrostatic interaction of poly(*N*-ethyl-3-vinylimidazolium bromide);^45^ (b) π–π stacking of polythiophene.^57^ Different parts of chemical structures are coded with the same colors as their corresponding strings.

In general, atom indexing is used to specify a range of atoms participating in a delocalized bond. For polyelectrolytes, if the charge participating in an electrostatic bond is delocalized across a group of atoms (see Fig. 3), the key/value pair, “*ne* = …”, is required, indicating the total delocalized charge. To avoid ambiguity in counting atoms for delocalized bonds, all annotation expressions for non-covalent bonding should be anchored to the right of the last atom involved, with the index 1 being assigned to that atom. Since any chemical bonds are interactions between atoms, only atom characters need to be indexed and counted here. Given that BigSMILES is a string-based representation of polymers, this results in increasing indices for atoms towards the left side of the string as illustrated below:
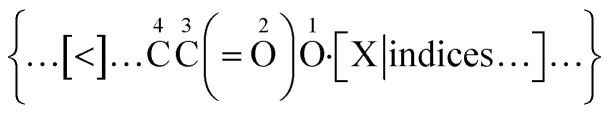


Note that atoms and ions of multiple characters, enclosed in square brackets under SMILES syntax, for example [Br−], are indexed as a single character.

The stacking interactions between extended, delocalized π systems are another important class of noncovalent interactions that involves groups of atoms.^56,57^ This type of interaction requires the same annotation syntax as delocalized charges (in Fig. 3a) to denote the set of atoms involved: with the annotation expression anchored to the last atom involved in the stacking, the bonds are denoted by the descriptor $: and the associated index range *x* ∼ *y* as illustrated in Fig. 3b. More representative examples of π–π stacking in polymeric systems can found in Fig. S12 and S13 in the ESI.†

### 2.4 State of non-covalent bonds

**Fig. 4:**
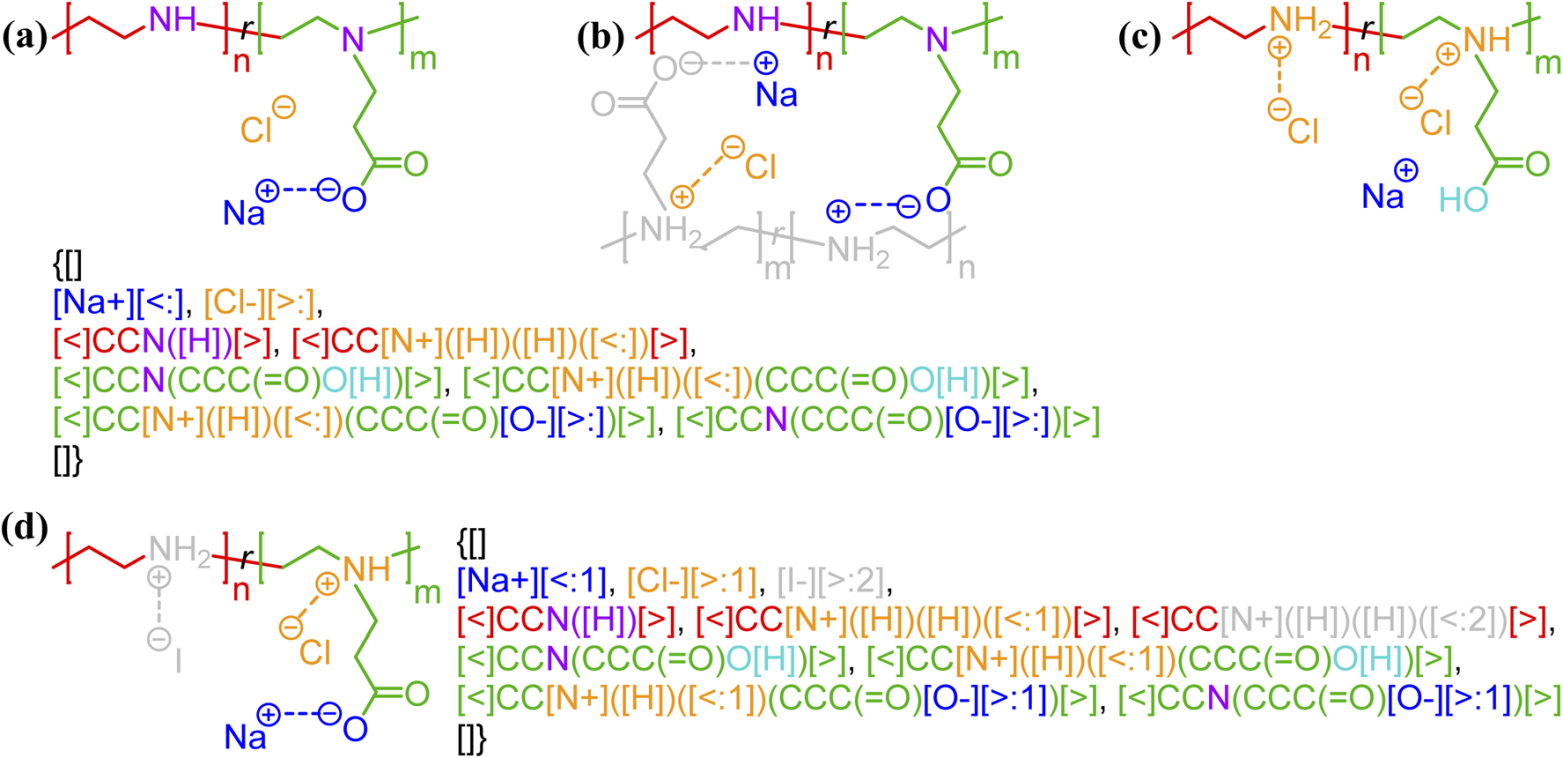
Different states of zwitterionic polyelectrolytes^62^ (methyl-ester functionalized linear poly-ethylenimine, LPEI) with (de)protonation state and the resulting electrostatic bonds enumerated to represent the system being at a specified (a) high and (b) moderate pH; and (c) low pH with depletion of salt as well as having (d) specified secondary ammonium salt/moiety (with iodide counterion ions) along polymer backbone. Panels a, b, and c are all represented by an identical non-covalent BigSMILES. Note that in the above figure, different parts of chemical structures are coded with the same colors as their corresponding strings.

Non-covalent bonding is strongly affected by the environment in which a polymer exists, so it is important to have flexibility to note these differing interactions. Taking the zwitterionic polyelectrolytes in Fig. 4 as an example, the polymer can be positively charged, negatively charged, neutral, or zwitterionic depending on the pH of the system. At intermediate pH, physical gels can be formed through an undetermined number of ion pairs between oppositely charged groups in the polyelectrolytes. Regardless of the exact physicochemical state of the polymer, the non-covalent BigSMILES language adopts the philosophy of representing all possible non-covalent bonds under consideration in the system. To avoid devising sophisticated notation for conditional bonds, this is achieved by enumerating the donor/acceptor state of each monomer in each block. This concept of multiple competing bonding partners is illustrated in Fig. 4. With charges and hydrogen atoms written explicitly for (de)pronated atoms, the same string (with color coding) can be used to represent all different bonding states. In case of multiple counterions in the system, specific electrostatic interactions (possibly due to the steric effects) can also be accounted for with bond indexing. As shown by Fig. 4d, the electrostatic interaction between an iodide anion and a quaternary anime cation is specified by having a different bond indexing (*i.e.*, [<:2], [>:2]) from the non-specified ones (*i.e.*, [<:1], [>:1]). Thus, unlike the covalent bonds, annotation on a non-covalent bond is only viewed as a potential connection. The probability of such a connection must be specified *via* bond indexing (see Fig. S3 in the ESI†) with an associated data structure where the relative abundance of each of bonding state can be included. Additional examples can be found in Fig. S1 and S2 in the ESI.†

### 2.5 Annotating groups of non-covalent bonds and correlated binding

**Fig. 5:**
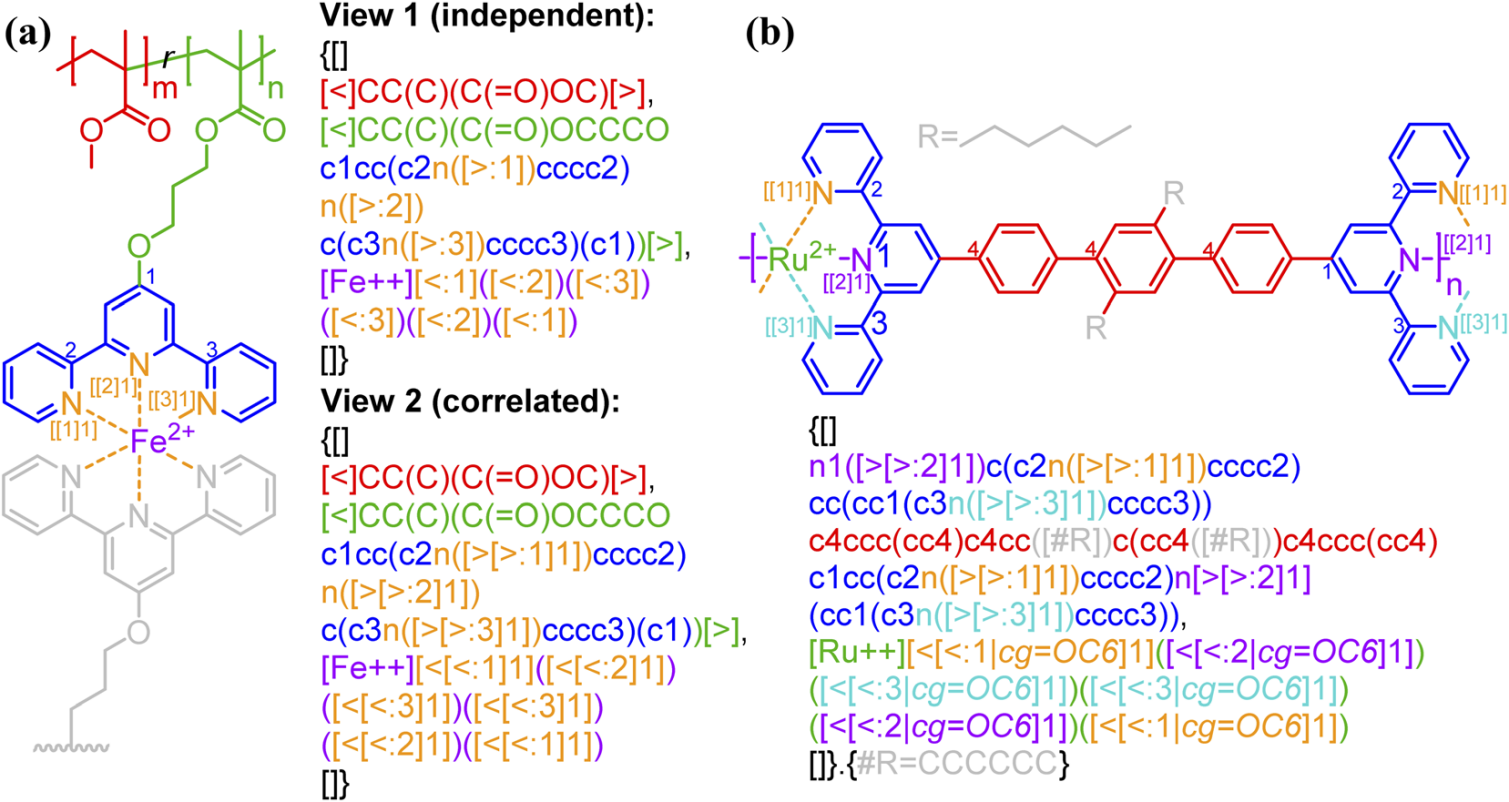
Illustration of how connectivity patterns are distinguished by the nested indexing on (a) non-correlated (view 1) *vs.* correlated (view 2) syntactic representations for metal–ligand complexation of Fe^II^-terpyridine functionalized methyl methacrylate copolymer;^54^ (b) coordination polymer (Ru^II^-4,4′′-bis(2,2′:6′,2′′-terpyridine)-2′,5′-dihexyl-*p*-terphenyl)^53^ with optional key/value (*i.e.*, *cg*) to denote the coordination geometry. Note that in panel b the syntax {…[#R]…}.{#R = …} is used to simplify the representation of alkyl side group with pseudo atom [#R]. Different parts of chemical structures are coded with the same colors as their corresponding strings. The single integers without brackets “*i*” are recursive nodes for cyclic structure, while the integers enclosed with two layers of square bracket “[[*i*]*j*]” are the indices of annotated groups of bonds.

Bond descriptors can be appended with a positive integer (*i.e.*, [$*i*], [>*i*], and [<*i*], *i* = 1, 2, …), to distinguish between different sets of connections within the same string. In the case of complex connectivity patterns across RUs, the nested indexing formalism […[…*m*]*n*] designed for ladder polymers can be adopted to represent metal–ligand complexation. A nested notation groups multiple specific bonds indexed in the inner square brackets, *i.e.*, […[…*m*]…] and *m* = 1, 2, …, into combined connectivity patterns denoted by those in the outer square brackets, *i.e.*, […[…]*n*]. Coordination polymers and metal organic frameworks (MOFs),^53,54^ through the descriptors <: and >:, may also be represented as either independent or correlated bonds; both of these perspectives are illustrated in Fig. 5a on the same system. Generally, these systems are represented with highly correlated bonding patterns. It is worthwhile to note that the above syntax cannot account for the subtlety that is not typically depicted in chemical structure-based representations, for instance the metal atom that can either be an acceptor or a donor.

By having metal ions as multivalent linkers and small-molecule ligands as RUs, the kinetically labile but thermally stable metal–ligand complexations can also be applied to make supramolecular polymer backbones (Fig. 5b). In the illustrated example, a single [Ru++] ion binds to two terpyridine ligands from both sides with six nitrogen atom sites in total to form an assembly. An optional key/value pair, “*cg* = …” (with possible values given in Table 1 and illustrated by Fig. S8 in the ESI†) can be employed to denote the coordination geometry. Note that more examples for the annotation of metal–ligand complexation can be found in Fig. S7–S9 in the ESI,† including examples illustrating supramolecular networks.

**Fig. 6:**
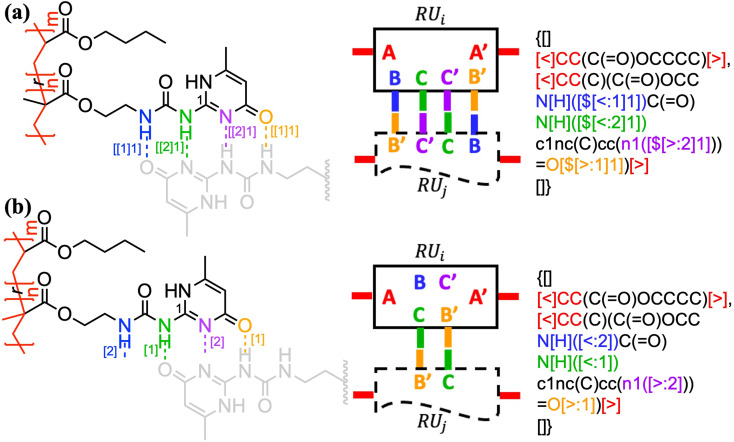
The indexing of non-covalent BigSMILES to annotate poly(*n*-butyl acrylate-*co*-UPy) polymers representing (a) fully correlated and (b) independent connectivity patterns.^52^ From left to right of each figure are the detailed chemical structures, the generic connectivity patterns between RUs, and their associated BigSMILES syntax respectively.

The above formalism can be readily extended to represent different binding patterns of multiple donors and multiple acceptors with generic connectivity, as shown in Fig. 6.^52^ The difference between Fig. 6a and b lies in whether the polymer backbone AA′ (colored by red) and two lateral hydrogen bonding pairs of UPy (Ureidopyrimidinone), *i.e.*, BB′ (colored by blue and orange) and CC′ (colored by purple and green), need to be formed in a correlated (Fig. 6a) or fully independent (Fig. 6b) manner: by nesting individual bonds into an indexed group, *i.e.*, […[…]1], such that each group represents a combined connection, stochastic bonding descriptors of the same group (as indicated by the same outer indices for AA′, BB′, and CC′ bonds in Fig. 6a), shall form in parallel with identical connectivity. To denote the fully independent bonding of two donor and acceptor pairs, their indices need to be assigned differently to represent distinct connectivity patterns (see the different indices for BC′ and CB′ bonds in Fig. 5b). A more complex example can be found in Fig. S3 in the ESI.†

## 3 General discussion

Since rigorous classification of different types of bonds is often difficult (for instance, ferrocene-like delocalized bonds as illustrated in Fig. S10 and S11 in the ESI†), the annotation formalism introduced above treats all types of non-covalent bonds with a universal format. Features (bond indexing, delocalized bonding, nested bonds for correlated bonding in ladder-type interactions) illustrated for one type of bond are uniformly applied for other types of bonds. The following summarizes important considerations when applying the above syntax to annotate different non-covalent chemistries:

### 3.1 Hydrogen notation

A hydrogen atom participating in a non-covalent bond needs to be written explicitly (*i.e.*, enclosed in square brackets, see the hydrogen of the hydroxyl group in Fig. 2c and the protonation of the amino group in Fig. 4 as examples) when it participates in annotated non-covalent bonds, such as in H-bonding and electrostatic interactions.

### 3.2 Layers of stochasticity

All non-covalent interactions are viewed as potential bonds whose stochasticity is naturally distinct from covalent ones as well as other non-covalent bonding of different category (for instance, electrostatic *vs.* hydrogen bonding). In addition, non-covalent interactions can occur between different polymers while the covalent ones are always restricted to local annotation of atoms.

### 3.3 Directionality

Unless being specified by the user, the overall directionality of a nested group (*i.e.*, the outer bond descriptors) is set to be the same as that of the inner bond under the lowest index: *i.e.*, [<[<:1…]…], [>[>:1…]…], [$[$:1…]…].

### 3.4 Uniqueness of key and index

In each set of non-covalent BigSMILES strings, both the index and key of the bonding descriptors need to be non-redundant for a clear and compact translation of the annotation. A single bond descriptor cannot be associated with two different bond types, and the same bonds cannot have different indices.

### 3.5 Semi-flexibility in format

Although the overall annotation format that begins with a bond descriptor and ends with key/value pairs needs to be enforced, most attributes (*i.e.*, bond and atom index as well as key/value pairs) are optional. The minimum requirement for a valid annotation is to have a non-covalent bond descriptor enclosed by square brackets.

### 3.6 User-orientated features

In close analogy to adjectives in a natural language, users may create their own keys and values that do not conflict with existing grammatical elements. Although the number and the type of key/value pairs are not restricted in a typical annotation expression, non-covalent BigSMILES is not devised to hold property information (such as bond strength, solubility) for polymers, which must be stored in an attached data structure/model. For best practice, only a minimal number of key/value pairs (see Table 1) that are intrinsic to the non-covalent bonding is allowed in the annotation expression.

### 3.7 Model independence

Although the notation is devised on the electronic nature of non-covalent bonds, it cannot surpass the limit of valence-based representation of chemicals. Non-covalent BigSMILES is equivalent to a string based ChemDraw figure, representing chemical structure with interactions the users choose to annotate. It reflects the bonding patterns of the most relevance among all possible interactions as identified and characterized by the user.

A key challenge in any non-covalent bond formalism is choosing which interactions to annotate. The philosophy taken with non-covalent BigSMILES is that the user may specify relevant interactions using annotations, but the interactions specified in the structure will not be a comprehensive list of all possible interactions. Because non-covalent bonds must be enumerated, BigSMILES only works for those interactions that are specific; nonspecific forces such as van der Waals interactions are difficult to annotate. Therefore, it is anticipated that non-covalent notation will be principally useful in annotating strong, dominant non-covalent interactions that play a critical role in the properties of a molecule, such as those that lead to many types of gelation and supramolecular self-assembly as illustrated in the examples above. While materials under different conditions may have different non-covalent representations, they will share a common covalent representation. By properly parsing a non-covalent BigSMILES string, it is possible to perform functions such as search in a way that is robust to variations in non-covalent annotation.

## 4 Towards annotating biopolymers and supramolecular assemblies

**Fig. 7:**
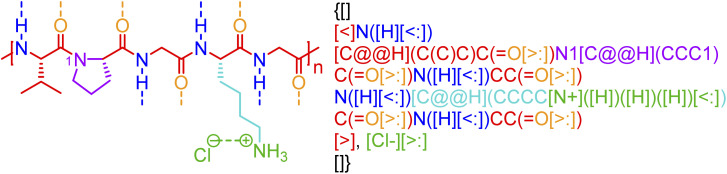
Non-covalent BigSMILES syntax is used to represent elastin-like polypeptide (ELP). Note that in the above figure, different parts of chemical structures are coded with the same colors as their corresponding strings. The single integers without brackets “*i*” are recursive nodes for cyclic structures. Note that syntax regrading chirality (C@H, C@@H) is directly borrowed from SMILES; additional examples and detailed explanations on the use of the above syntax can be found in the literature.^39^

Although biopolymers often may be considered as sequence-defined macromolecules and therefore non-stochastic by nature, they are often used in hybrid systems that have stochasticity, such as glycosylated proteins,^63^ protein-polymer bioconjugates,^64^ bio-inspired materials,^65^ and peptide- and nucleic acid-containing gels.^66,67^ Furthermore, in many cases, the non-covalent bonding in a biopolymer is stochastic. Biomaterial classes, such as consensus-repeat proteins,^68,69^ that are often used in protein materials, can be represented through non-covalent BigSMILES by including the relevant annotations for electrostatic interactions and hydrogen bonding associated with each amino acid residue. An example of this with an elastin-like polypeptide (ELP) is shown in Fig. 7. Computer program that accepts amino acid sequences and output BigSMILES strings with their non-covalent interactions have been built and elaborated in the ESI.† Although the annotation of the above classes of non-covalent chemistry in proteins or polypeptides is alone insufficient for describing higher-order protein structure (additional data would be required in an associated data structure), this level of annotation describes protein/polypeptide interactions to a similar degree to that seen in synthetic polymer materials.

So far, a wide variety of examples (including those in the ESI†) have been illustrated where diverse non-covalent bonds are depicted with their corresponding syntax. This expansion of BigSMILES also opens opportunities for the meaningful annotation of supramolecular assemblies as listed in Table 2, which includes but not limited to multi-component coacervates and gels (see Fig. S6 in the ESI†), ferrocene-like delocalization (see Fig. S10 and S11 in the ESI†), host–guest interactions as well as polycyclic aromatic-based chain folding (see Fig. S14–S16 in the ESI†). As demonstrated by Table 2 with more examples elaborated in the ESI,† annotation of non-covalent interaction is critical to properly describe these classes of molecules.

**Table 2:** Examples of annotated supramolecular complexes assembled with non-covalent interactions

Class	Example	Class	Example
Multi-component complexation	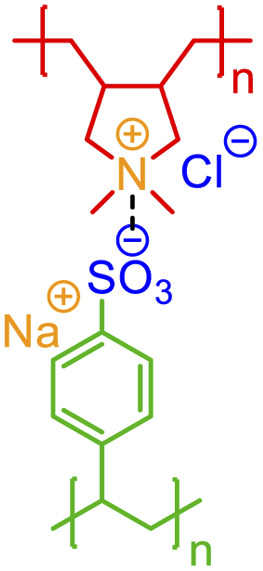	Host-guest interaction	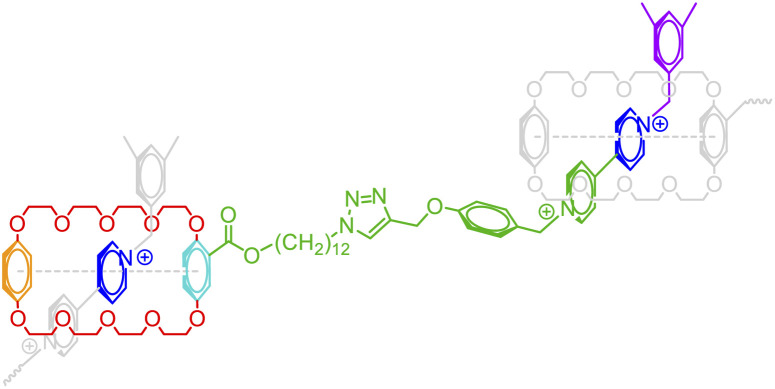
Ferrocene-like delocalization	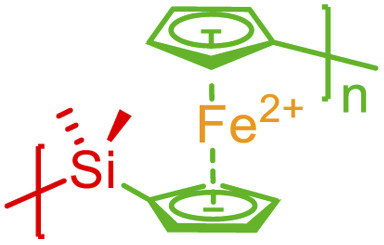	Aromatic-based chain folding	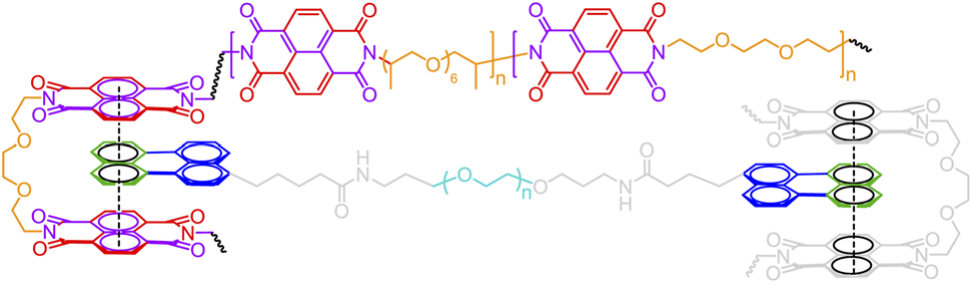

## Conclusion

Here, an annotation method is developed to extend BigSMILES to include non-covalent interactions related to polymer and supramolecular assemblies. Non-covalent BigSMILES extends from the framework of the original BigSMILES syntax, with devised formalisms that reasonably depict many types of supramolecular interactions within the same system, including polyelectrolytes, hydrogen-bonded polymers, metallogels, polycyclic aromatic polymers with pyrene-based chain folding, and supramolecular polymers with host-guest interactions. Further extension of non-covalent BigSMILES to other related chemistries is foreseeable. The general formalism used for annotating these non-covalent interactions starts with a bond descriptor of donor–acceptor character that captures Lewis acid/base and electrostatic interactions, and adds minimal key/value pairs, whose presence in the annotation expression closely resembles the role of adjectives in a natural language. Most of the challenges associated with the bond connectivity are solved by appending a proper set of indices.

Because it is a string-based identifier with atomistic resolution, non-covalent BigSMILES provides a compact representation of the covalent and non-covalent stochastic graphs that make up polymer and supramolecular materials. Non-covalent BigSMILES therefore captures many of the variables in molecular fingerprints used in machine learning; however, the human readability and simplicity of the strings require that only information intrinsic to the chemical structure is annotated. For best practice, users are encouraged to defer any property data to a separate data structure/model, such as polyDAT, where the specific states of the system can be saved. The variety of non-covalent BigSMILES strings reported here shows that the above syntax produces a faithful description of diverse chemistries and polymeric systems. Since users are allowed to formulate their own context-annotated features, *i.e.*, key/value pairs, this language offers a promising approach for encoding complex supramolecular architectures and biopolymers. The generality of the donor–acceptor principle used in the non-covalent BigSMILES syntax also makes it able to represent other less-common supramolecular interactions, such as halogen bonding or frustrated Lewis pairs, providing a highly generalizable approach to representing non-covalent chemistry.

## Author contributions

W. Z., T. S. L. and B. D. O. conceived of the idea. W. Z. developed the methodology. A. M. M., Y. Y., S. C. M., and M. M. C. analyzed and validated the methodology on four major classes of non-covalent chemistry. N. J. R., H. K. B., M. A. M., and C. S. C. analyzed and validated the methodology on another two classes of non-covalent chemistry. J. A. K., S. L. C., A. N., and J. S. M. edited the manuscript. W. Z. and B. D. O. wrote the manuscript.

## Conflicts of interest

There are no conflicts to declare.

## Supplementary Material

SC-013-D2SC02257E-s001
