# Supplementary material for: Extending BigSMILES to non-covalent bonds in supramolecular polymer assemblies
Source: Chem Sci. 2022 Sep 15;13(41):12045–55. doi: 10.1039/d2sc02257e (PMC9600227; doi:10.1039/d2sc02257e)
Supplement: SC-013-D2SC02257E-s001 [file SC-013-D2SC02257E-s001.pdf]

Electronic Supplementary Material (ESI) for Chemical Science.
This journal is © The Royal Society of Chemistry 2022

Supplementary information

Extending BigSMILES to Non-Covalent Bonds in Supramolecular Polymer Assemblies

Weizhong Zou[1], Alexis Martell-Monterroza[2], Yunxin Yao[3], S. Cem Millik[4], Morgan M. Cencer[5], Nathan J. Rebello[1], Haley K. Beech[1], Melody A. Morris[1], Tzyy-Shyang Lin[1], Cleotilde S. Castano[7], Julia A. Kalow[2], Stephen L. Craig[3], Alshakim Nelson[4], Jeffrey S. Moore[5,6], Bradley D. Olsen[1‡]

1. Department of Chemical Engineering, Massachusetts Institute of Technology, Cambridge, MA 02142
2. Department of Chemistry, Northwestern University, Evanston, IL 60208
3. Department of Chemistry, Duke University, Durham, NC 27708
4. Department of Chemistry, University of Washington, Seattle, WA 98195
5. Department of Chemistry, University of Illinois at Urbana-Champaign, Urbana, IL 61801
6. Beckman Institute for Advanced Science and Technology, University of Illinois at Urbana-Champaign, Urbana, IL 61801
7. Roxbury Community College, Boston, MA 02120
‡Correspondence: email: bdolsen@mit.edu, phone: (617) 715-4548.

Table of Contents

Polyelectrolytes....................2
Hydrogen bonding....................4
Polyelectrolyte and hydrogen-bonded networks....................6
Coordination polymers, polyMOFs and polyMOCs....................7
Ferrocene-like delocalization....................10
π-π stacking....................12
Chain folding & host-guest interactions....................14
Biopolymers....................16
References....................18

In this document, more sophisticated examples are shown for annotating non-covalent chemistry in polymers. Among them, polymers or supramolecular assemblies containing single or multiple types of non-covalent bonding descriptors are represented under the categories of polyelectrolytes,[1-10] hydrogen bonding,[11-17] coordination polymers, polyMOFs and polyMOCs,[18-25] ferrocene-like delocalization,[26-34] π-π stacking,[35-43] chain folding and host-guest interactions,[44-49] as well as biopolymers.[41, 50] Given that non-covalent BigSMILES is equivalent to a string-based ChemDraw figure, which represents the bonding patterns of the most relevance among all possible interactions as being identified and characterized by the user, the following examples demonstrate the capability of the developed syntax to accommodate the broad scope of chemistry in polymer science.

Polyelectrolytes

More examples for annotating polyelectrolytes are shown here. In Fig. S1a and b, polycation and polyanion copolymers are represented with charged comonomers binding to counterions from added salts. For zwitterionic polyelectrolytes, the relative abundance of positively and negatively charged counterions can vary depending on pH (Fig. S1c and d). As discussed in the main text, this uncertainty can be avoided by deferring the specification on bonding state to a data model, which greatly simplifies annotations on a great variety of zwitterionic polyelectrolytes as illustrated in Fig. S2. Note that only the depronated state of sulfonate groups is represented in the following examples, given it must be in extreme conditions to have both pronated and depronated states for strong polyelectrolytes where a polymer is usually completely ionized.

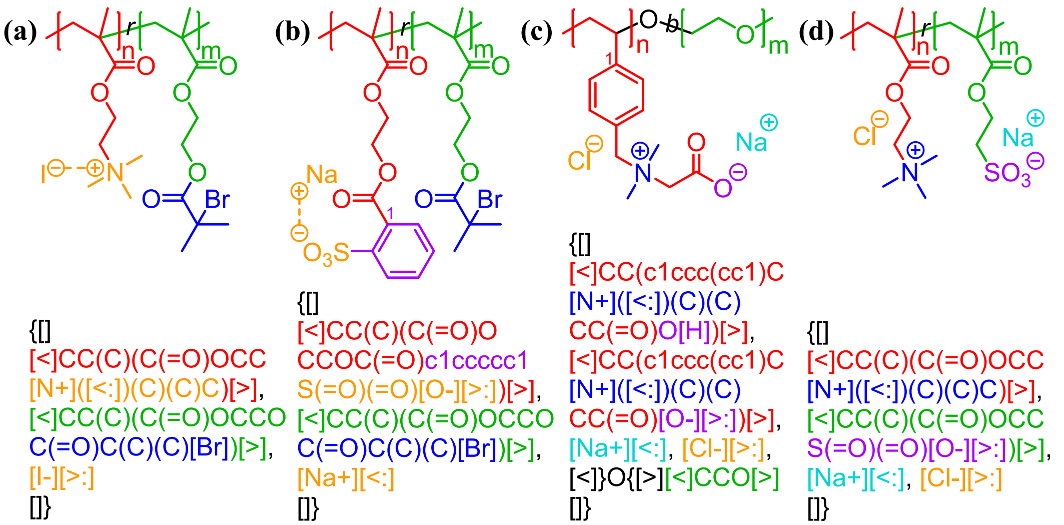

Figure S1. Annotation of electrostatic bonds in polyelectrolyte copolymers with examples (a) strong polycation DMA-HEMA (dimethylacetamide-(hydroxyethyl) methacrylate) random copolymer esterified with excess BIBB (2-bromoisobutyryl bromide) as well as quaternized by excess methyl iodide;[51] (b) strong polyanion poly-HEMA esterified by BIBB and SBA (2-sulfobenzoic acid cyclic anhydride) in sequence;[51] and zwitterionic polyelectrolytes of a general bonding state with positively and negatively charged group in the same and different monomers: (c) betaine functionalized vinylbenzyl chloride; (d) sulfoethyl methacrylate and (methacryloyloxy) ethyl trimethylammonium chloride.[52-53] Note that in the above figure, different parts of chemical structures are coded with the same colors as their non-covalent BigSMILES strings.

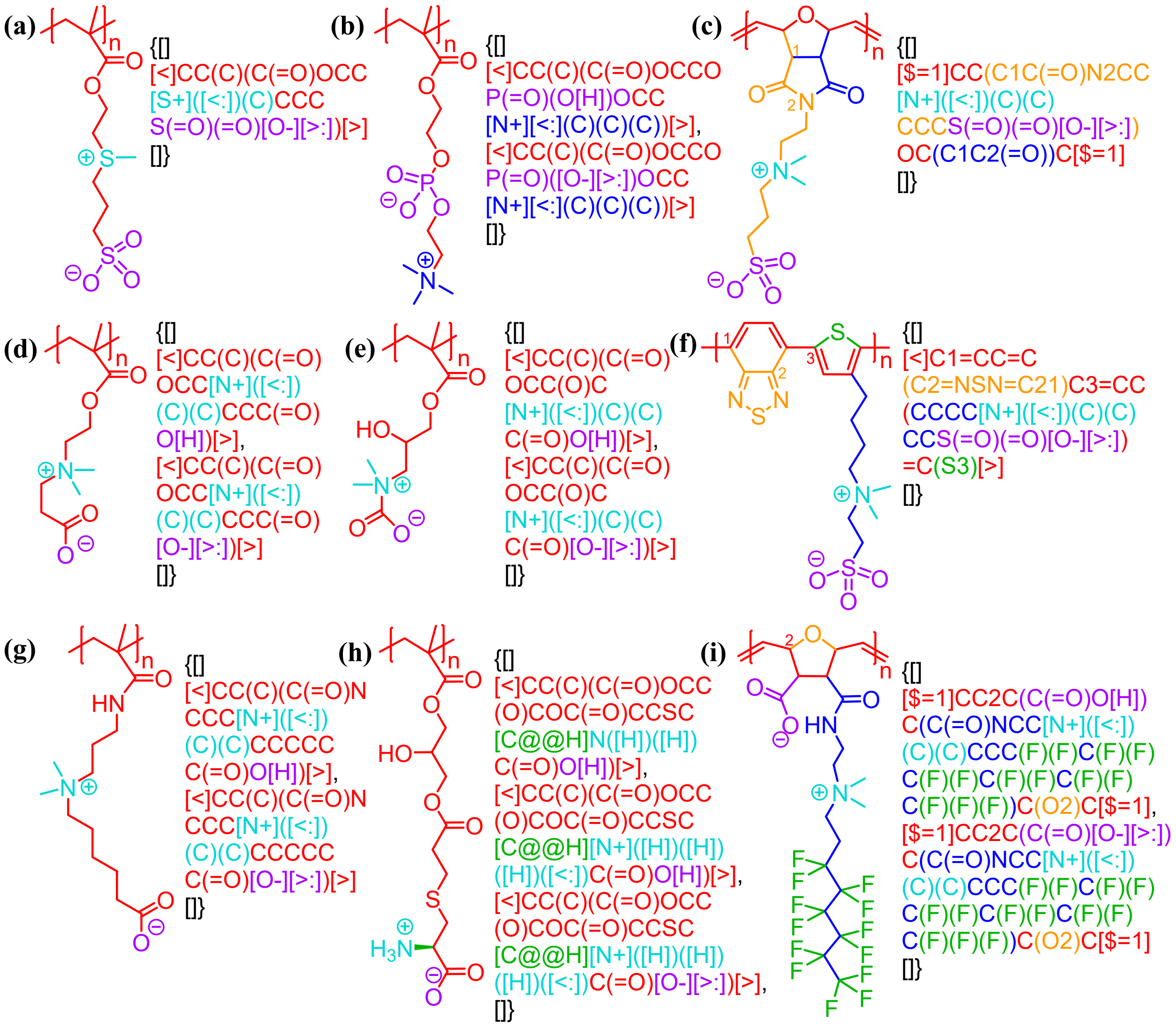

Figure S2. Examples of zwitterionic polyelectrolytes represented by general state of electrostatic bonding. (a) polymeric sulfothetin, monomer of 3-(methyl{2-[(2-methylacryloyl)oxyl]ethyl} sulfaniumyl) propane-1-sulfonate;[3] (b) poly(2-methacryloyloxyethyl phosphorylcholine);[4] (c) polynorbornene based sulfobetaines;[5] (d) poly(carboxybetaine methacrylate);[6] (e) poly(carboxybetaine methacrylate);[7] (f) thiophene based conjugated polyzwitterions;[8] (g) polycarboxybetaine acrylamides;[2] (h) poly(cysteine methacrylate);[9] (i) fluorinated norbornene based polybetaine.[10] Note that in the above figure, different parts of chemical structures are coded with the same colors as their non-covalent BigSMILES strings. The single integers without brackets "i" are recursive nodes for cyclic structure.

Hydrogen bonding

To complement those included in the main text, additional examples to illustrate the annotation syntax for H-bonding are given in Figs. S3 to S5. In Fig. S3, the capability of our annotation syntax is demonstrated by distinguishing different connectivity patterns for polymers self-assembled via H-bonding. 1) The fully independent binding of two donor and acceptor sites (coded by orange, green, purple and blue in Fig. S3a) in each monomer contained by the same or different polymer requires no indexing on any hydrogen bond descriptor as any pairwise connection can potentially form. If a rigorous one-to-one binding must be specified across all monomers between closely

aligned polymer molecules, as depicted by Fig. S3b, both the covalent bonding descriptors along the polymer backbone and the lateral H-bonding in-between have to be indexed with the nested formulism, indicating that they have formed ladder-polymer-like assembly. When coherent binding only exists for a specified pair of donor and acceptor in the monomer, i.e., the scenario of Fig. S3c, the polymeric material in question is anticipated to maintain a network morphology with gel-like properties.

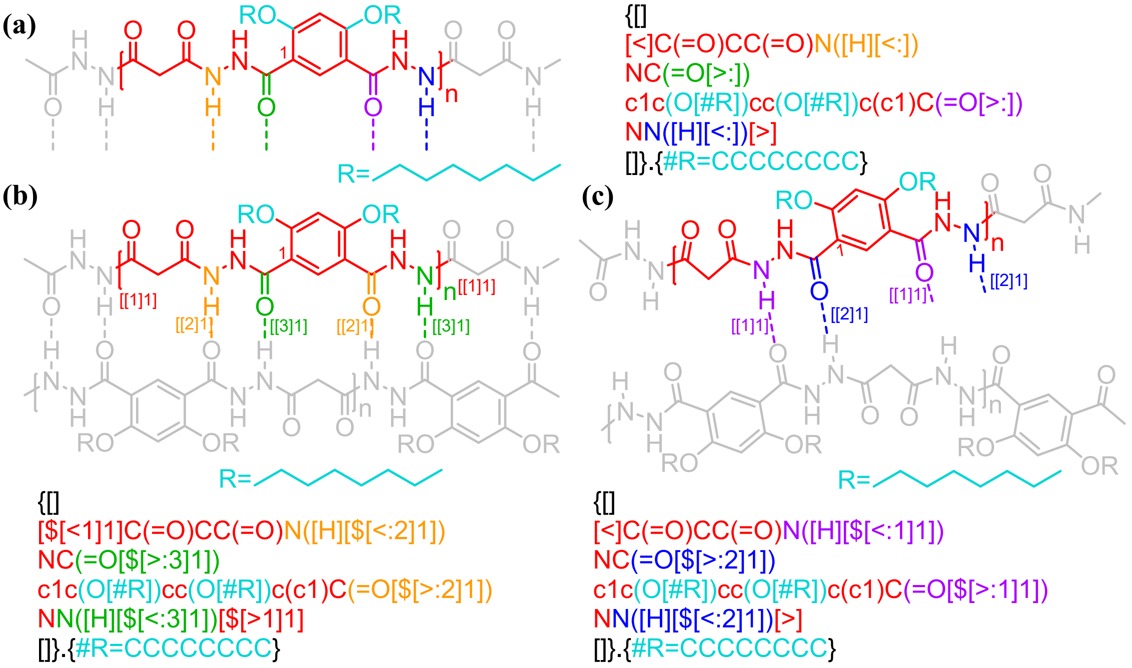

Figure S3. Different connectivity patterns of hydrazide derivatives[15] distinguished by the group indices on two lateral hydrogen bonds[15] with (a) fully independent; (b) rigorous one-to-one; and (c) specific pairwise binding. Note that in the above figure, different parts of chemical structures are coded with the same colors as their non-covalent BigSMILES strings.The single integers without brackets "i" are recursive node for cyclic structure, while the integers enclosed with two layers of square bracket "[[i]j]" are the indices of annotated groups of bonds.

Given the philosophy that an annotated non-covalent bond is only viewed as a potential connection, without indexing the hydrogen bonding in panel a of Fig. S3 accounts for both scenarios depicted in panel b and c. Such concept is further illustrated in Fig. S4, where the nested indexing formalism describes the correlated bonding of multiple donor and acceptor pairs. Physical gels are formed by hydrogen bonds with small molecule crosslinkers (in Fig. S4a) as well as with functional groups of (co)monomer (i.e., from the same polymer backbone in Fig. S4b and c). In Fig. S5, the same nested annotation as that of Fig. S4 is applied to represent the binding of Upy-like pairs when constructing both helical (panel a) and looped (panel b) architectures.

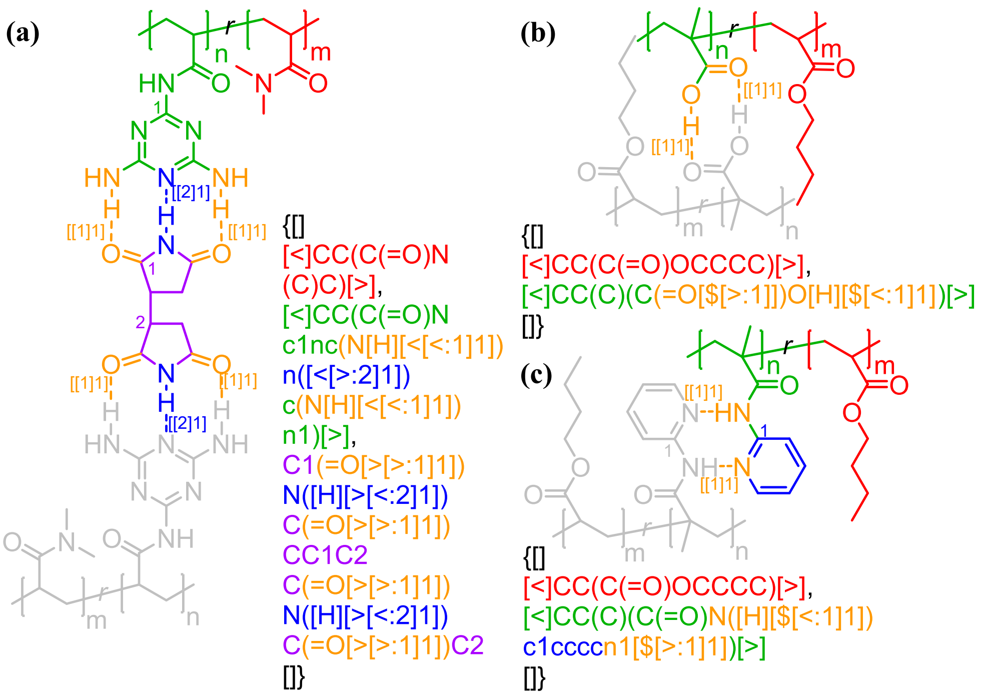

Figure S4. Annotation of physical gels formed by hydrogen bonds under a small molecule crosslinker (a) bis-maleimide crosslinked methacryl-succinimidyl functionalized poly(N-isopropylacrylamide),[13] and the same indexing of copolymer backbone (b) poly-(butyl acrylate)-co-(acrylic acid) and (c) poly-(butyl acrylate)-co-(acrylamidopyridine)[16] to reflect the connectivity patterns. Note that in the above figure, different parts of chemical structures are coded with the same colors as their non-covalent BigSMILES strings. The single integers without brackets "i" are recursive nodes for cyclic structure, while the integers enclosed with two layers of square bracket "[[i]j]" are the indices of annotated groups of bonds.

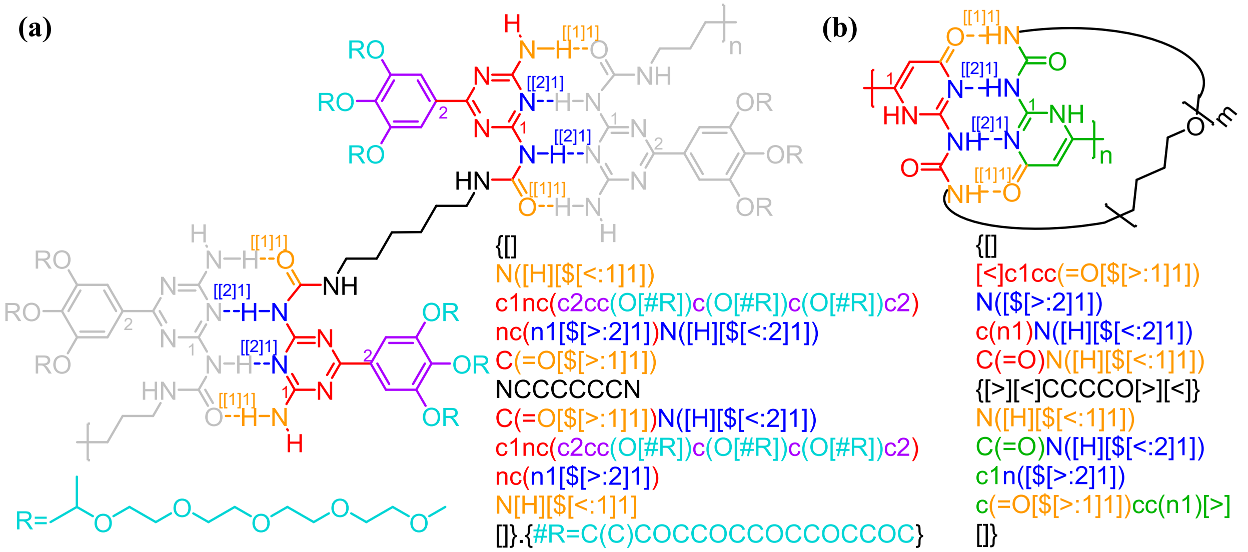

Figure S5. The use of non-covalent BigSMILES syntax to represent architecture assembled by H-bonding between pairs of Upy-like motifs: (a) helical (bifunctional ureido-s-triazines with penta-ethylene-oxide side chains)[17] and (b) looped (poly(tetramethylene glycol) of dimerized Upy ends)[11]. Note that in the above figure, different parts of chemical structures are coded with the same colors as their non-covalent BigSMILES strings. The single integers without brackets "i" are recursive nodes for cyclic structure, while the integers enclosed with two layers of square bracket "[[i]j]" are the indices of annotated groups of bonds.

Polyelectrolyte and hydrogen-bonded networks

For complicated multi-component systems, such as coacervate complexes and hydrogels, the overall system should be represented as a combination of non-covalent BigSMILES strings with bonding interactions between molecules. This formalism can be handled using additional pair of {...} as illustrated in Fig. S6. Unlike Fig. S1c and d, the association of polycation and polyanion in Fig. S6a (which exemplifies the construction of layer-by-layer assembly) occurs between different polymers with the bond descriptors of donor and acceptor of distinct stochasticity. To properly represent such "multi-component" systems, all interacting macromolecular components of strings shall be enclosed in [..., ...] to annotate the non-covalent binding of multiple polymers as being a supramolecular assembly. The resulting specification of the inter-component electrostatic bonds is borne out of the local BigSMILES strings that represent each component. This is different from covalent bonds, which are always local to a single BigSMILES string. Furthermore, as depicted in panels b and c, gels formed with hydrogen bond-based functional groups are annotated, which again requires the mixture formalism due to the correlated bonding of multiple components.

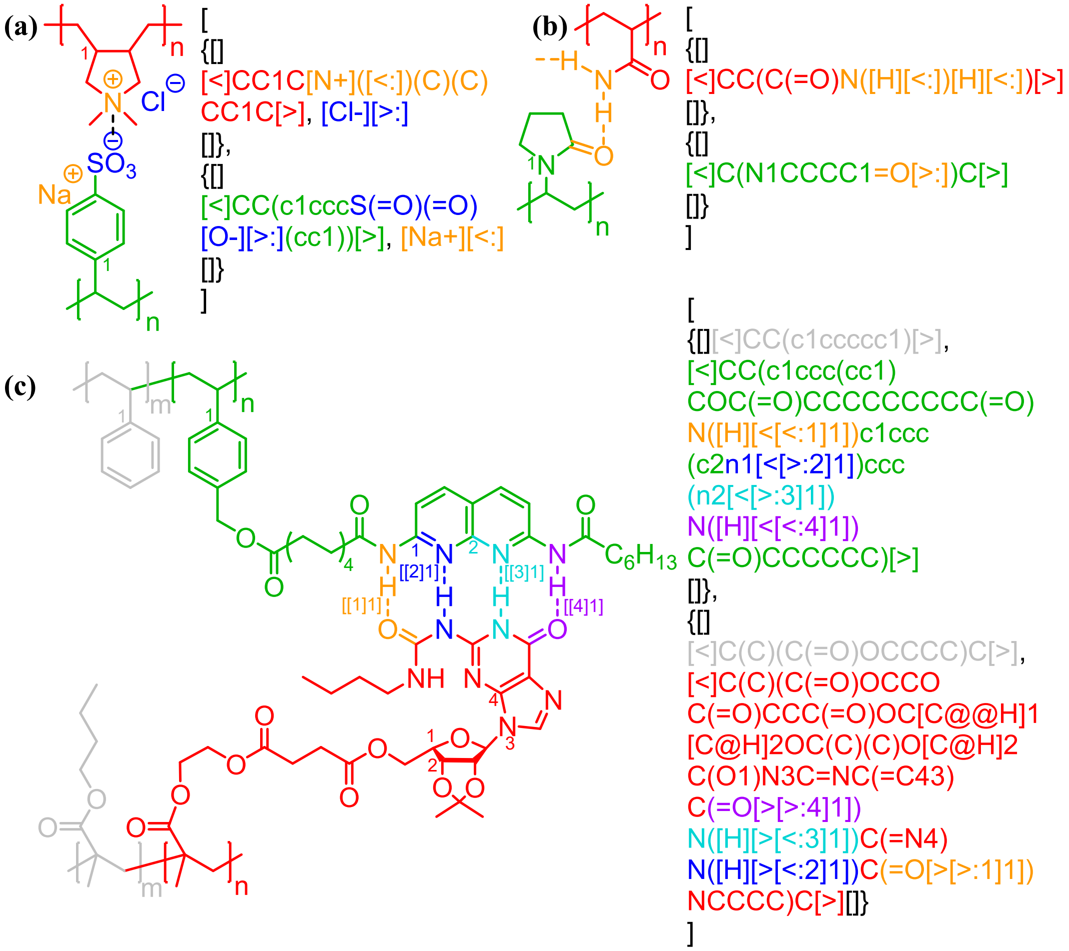

Figure S6. The annotation of coacervate (a) poly(styrenesulfonate) with poly(diallyldimethylammonium chloride),[52] and hydrogen-bonded networks: (b) polyacrylamide with poly(N-vinylpyrrolidone);[14] (c) 2,7-diamido-1,8-naphthyridine incorporated polystyrene with urea of guanosine incorporated poly(butyl methacrylate);[12] formed by electrostatic and hydrogen bonding of multi-component polymer mixtures. Note

that in the above figure, different parts of chemical structures are coded with the same colors as their non-covalent BigSMILES strings. The single integers without brackets "i" are recursive nodes for cyclic structure, while the integers enclosed with two layers of square bracket "[[i]j]" are the indices of annotated groups of bonds.

Coordination polymers, polyMOFs and polyMOCs

With metal ions and small molecule ligands as RUs (repeating units), coordination polymers, polyMOFs and polyMOCs can be properly annotated under the above syntax as shown in Fig. S7 and Fig. S8. The use of additional key/value pairs to annotate the coordination geometry and the number of electrons involved in metal ligand complexation is also illustrated in Fig. S8. Figure S9 shows representations of supramolecular networks for polymer ligands with metal ions acting as multivalent crosslinkers.

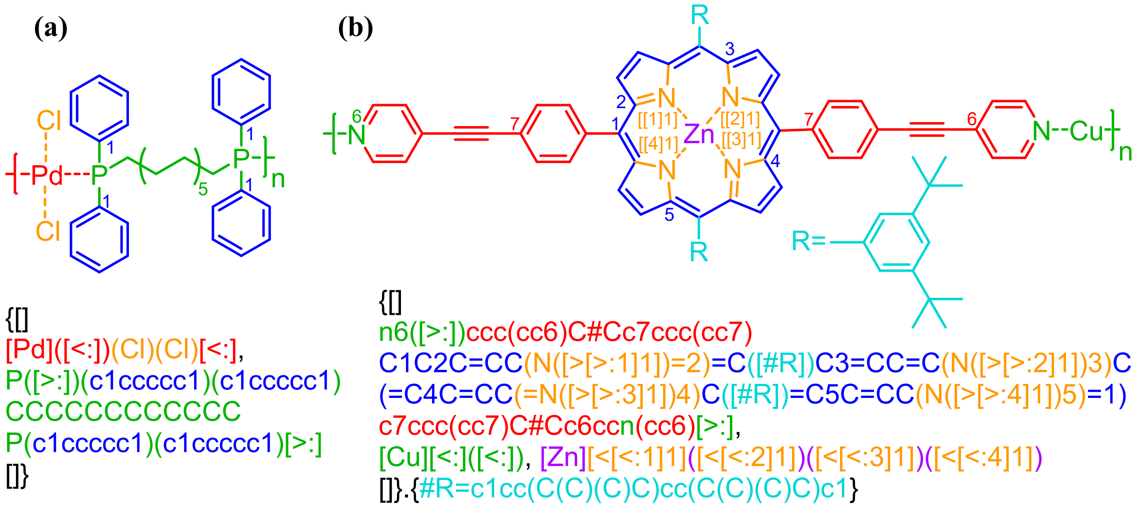

Figure S7. Examples showing proper annotation of coordination polymers. (a) Pd^{II}-alkyldicyclohexylphosphine;[24] (b) coor Cu^{II}-(phen-1,4-diyl-ethynyl-pyridyl) substituted porphyrin derivates.[19] Note that in the above figure, different parts of chemical structures are coded with the same colors as their non-covalent BigSMILES strings. The single integers without brackets "i" are recursive nodes for cyclic structure, while the integers enclosed with two layers of square bracket "[[i]j]" are the indices of annotated groups of bonds.

(a)

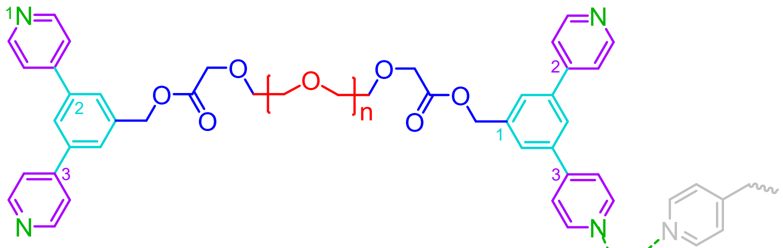

```
{[]
n1([>:])ccc(cc1)c2cc(cc(c3ccn([>:])cc3)c2)
COC(=O)COC{[>][<]COC[>][<]}COCC(=O)OC
c1cc(c2ccn([>:])cc2)cc(c3ccn([>:])cc3)c1,
[Pd++][<:|cg=TH4]([<:|cg=TH4])([<:|cg=TH4])([<:|cg=TH4])
[]}
```

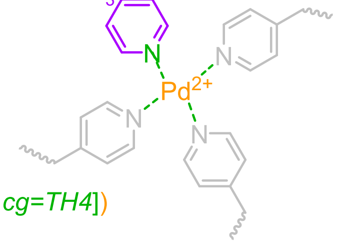

(b)

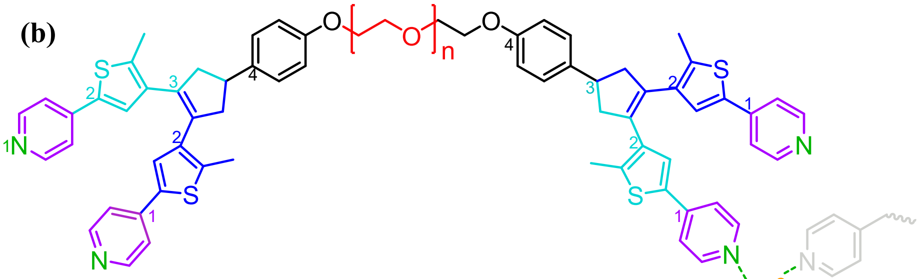

```
{[]
n1([>:])ccc(cc1)C2SC(C)=C(C=2)C3CC
(CC(=3)C2=C(C)SC(=C2)c1ccn([>:])cc1)
c4ccc(cc4)O{[>][<]CCO[>][<]}CCOc4ccc(cc4)
C3CC(=C(C3)C2=C(C)SC(=C2)c1ccn([>:])cc1)
C2=C(C)SC(=C2)c1ccn([>:])cc1,
[Pd++][<:|cg=TH4]([<:|cg=TH4])([<:|cg=TH4])([<:|cg=TH4])
[]}
```

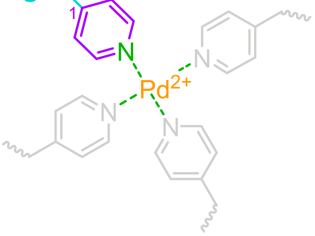

(c)

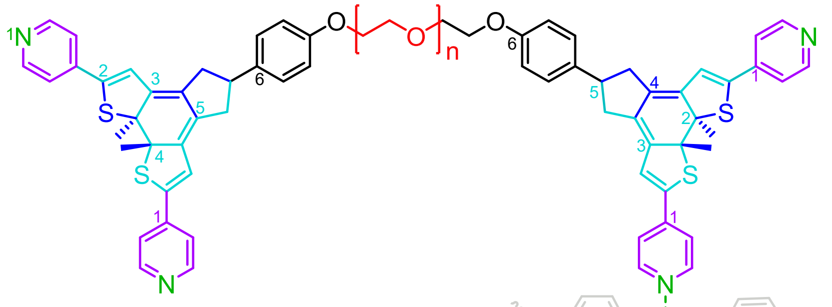

```
{[]
n1([>:])cc(cc1)C2=CC3C(S2)([C@@H])
C4([C@H])SC(c1ccn([>:])cc1)=CC4=C5CC(CC5(=3))
c6ccc(cc6)O{[>][<]CCO[>][<]}CCOc6ccc(cc6)
C5CC(C4C5)=C3C=C(c1ccn([>:])cc1)SC3([C@H])
C2([C@@H])C(=4)C=C(S2)c1ccn([>:])cc1,
[Pd++][<:|cg=TH4]([<:|cg=TH4])([<:|cg=TH4])([<:|cg=TH4])
[]}
```

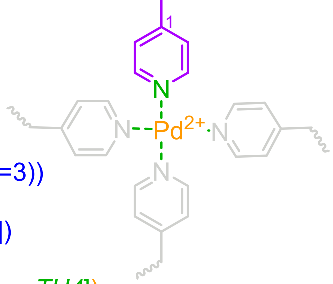

Figure S8. Annotation of supramolecular network formed by polymeric ligands and metal-organic cages (MOCs) as junctions with the use of an optional key/value pair to depict the coordination geometry involved. (a) Pd^{II}- para-bispyridyl terminated poly(ethylene glycol) ligands;[25] (b) and (c) Pd^{II}-bis-pyridyl dithienylethene terminated poly(ethylene glycol).[18] Note that in the above figure, different parts of chemical structures are coded with the same colors as their non-covalent BigSMILES strings. The single integers without brackets "i" are recursive nodes for cyclic structure, while the integers enclosed with two layers of square bracket "[[i]j]" are the indices of annotated groups of bonds.

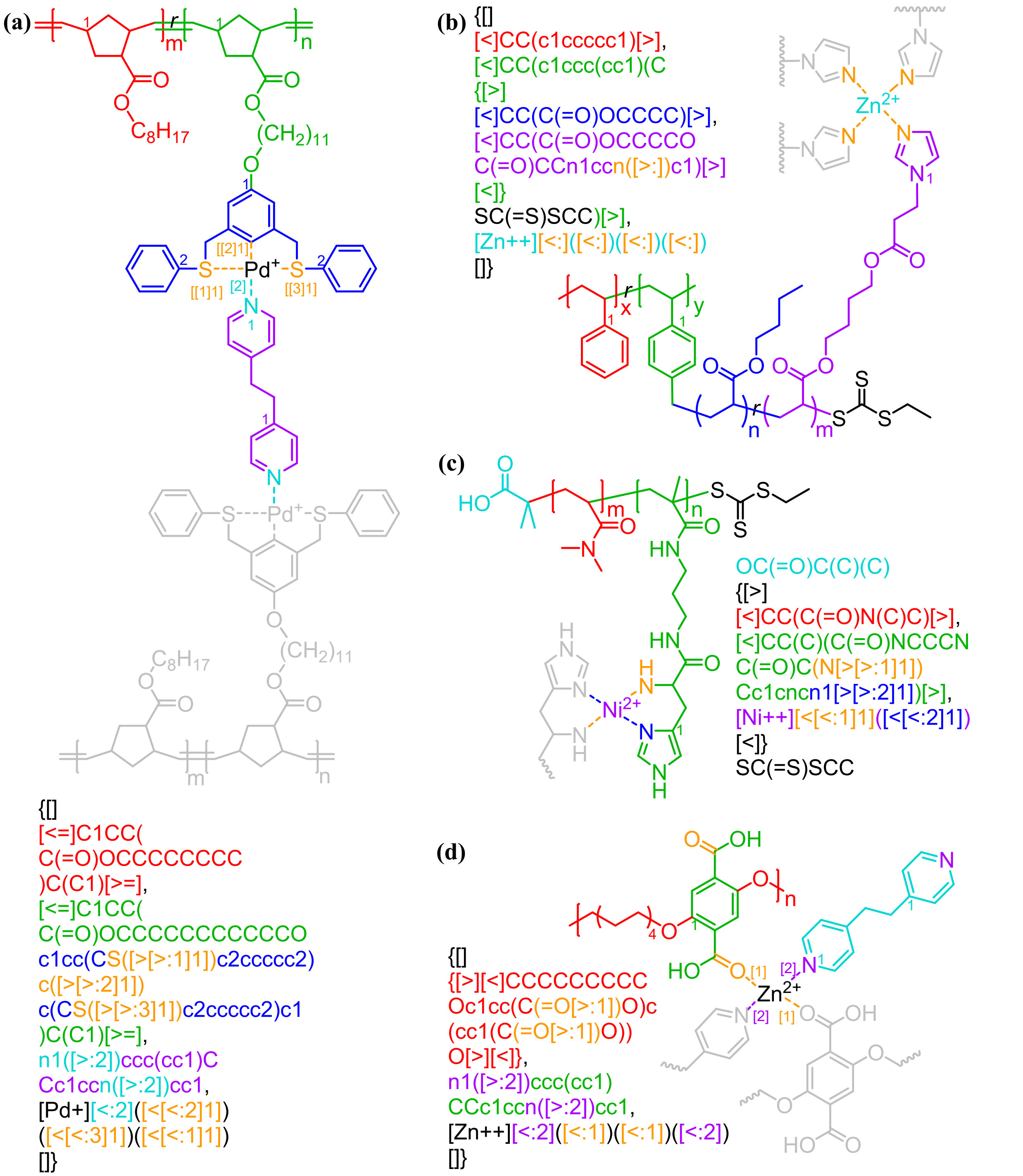

Figure S9. Annotation of supramolecular networks formed by metal polymer ligand complexation. (a) norbonene carboxylic acid ester based copolymer with palladated SCS pincer complexes coordinated to bispyridine;[23] (b) polystyrene based imidazole-containing brush polymer with Zn^{II};[22] (c) Ni^{II} coordinate with histidine-functionalized poly(N,N-dimethyl-acrylamide);[21] (d) coordination complex of Zn^{II} with co-ligands of 1,2-(4′,4′′-bipyridyl)ethane and poly(1,4-dihydroxyterephthalic ester).[20] Note that in the above figure, different parts of chemical structures are coded with the same colors as their non-covalent

BigSMILES strings. The single integers without brackets "i" are recursive nodes for cyclic structure, while the integers enclosed with two layers of square bracket "[[i]j]" are the indices of annotated groups of bonds.

Ferrocene-like delocalization

As a specific class of metal-ligand complexation, ferrocene-like delocalization involves interactions between a metal ion and a group of atoms with conjugated π-orbitals. By having the atom indices for delocalization (see Fig. 3) in the metal-ligand bond descriptors (i.e., View 2 of Fig. 5a in the main text), examples for polymers with such interactions are shown to be well represented here in Figs. S10 and S11.

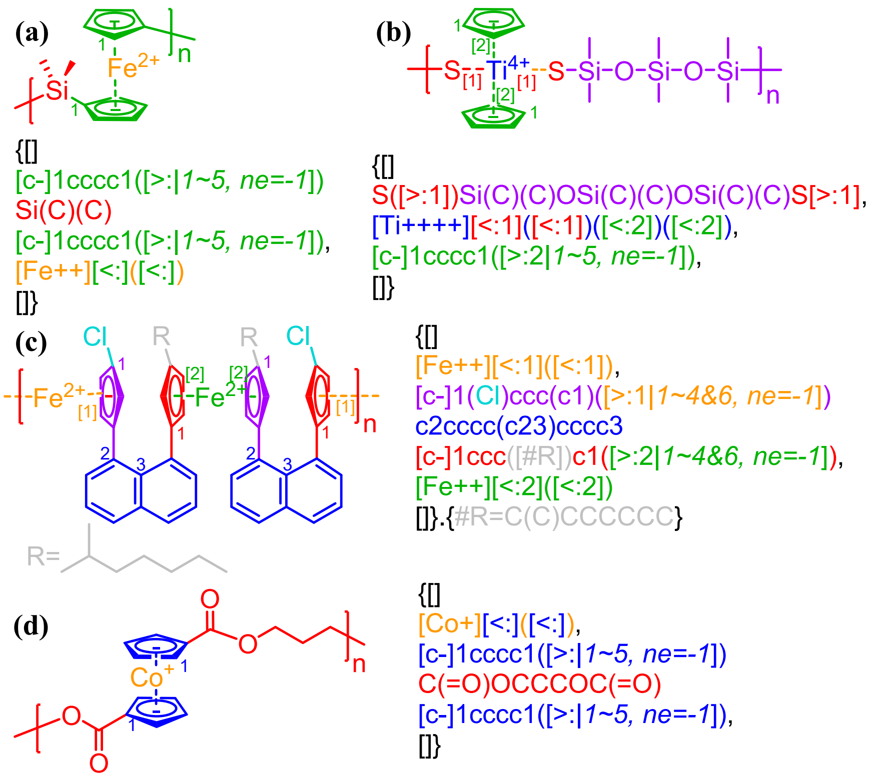

Figure S10. Annotation of polymer-like assemblies formed by ferrocene-like delocalization. (a) poly-ferrocenylsilane;[26] (b) poly(titanocenyl dimethylsiloxane disulfide);[29] (c) stacked ferrocenes of alkylated bis-(cyclopentadienyl) naphthalene;[27] (d) poly(1,4-butanediol 1,1'-dicarboxycobalticinium ester).[28] Note that in the above figure, different parts of chemical structures are coded with the same colors as their non-covalent BigSMILES strings. The single integers without brackets "i" are recursive nodes for cyclic structure, while the integers enclosed with two layers of square bracket "[[i]j]" are the indices of annotated groups of bonds.

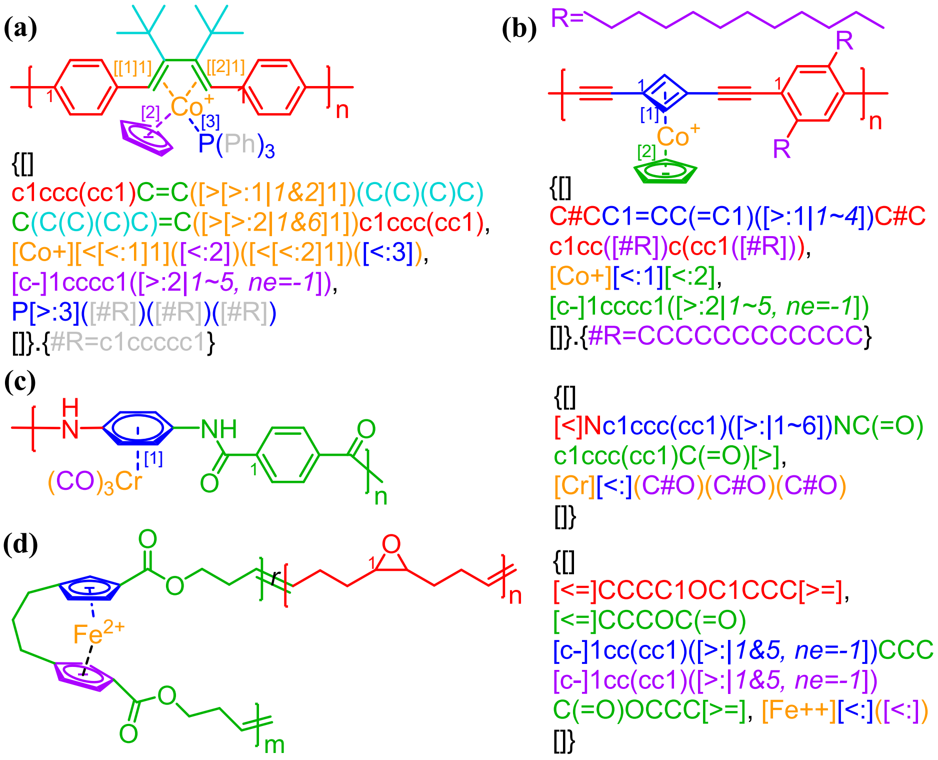

Figure S11. Additional examples showing annotation of polymers with ferrocene-like delocalization. (a) π-conjugated polymer of diphenyl-cobaltacyclopentadiene substitutes;[33] (b) polymer containing 2,5-(dialkyl)benzene and [1,3-(diethynyl) cyclobutadiene] cyclopentadienylcobalt moiety;[30] (c) complexation of poly (phenyleneterephthalamide Cr(CO)$_3$);[34] (d) copolymer of cis-[3]ferrocenophane macrocycle monomer with 9-oxabicyclo[6.1.0]non-4-ene (epoxy-COD).[32] Note that in the above figure, different parts of chemical structures are coded with the same colors as their non-covalent BigSMILES strings. The single integers without brackets "i" are recursive nodes for cyclic structure, while the integers enclosed with two layers of square bracket "[[i]j]" are the indices of annotated groups of bonds.

π-π stacking

More annotation examples for π-π stacking are included in the following figures. Figure S12 shows examples where small molecules are stacked to form polymer-like assemblies with the interacting atoms represented by their complementary indices via expression |*x~y*. In fact, there are three distinct formalisms for atom indexing: a) *x~y*, where integers *x* and *y* are the two inclusive boundary values for the range of indices, with the step size of 1; b) *x&y&z*, where integer *x*, *y*, and *z* are combined by "&" and are indices of individual atom characters to include for the annotation; or c) *x~y!m!n*, where atoms with indices in the range of integers from *x* to *y* are included except those of index *m* and *n* (the exclamation symbol "!" is equivalent to logical operator "not"). It is worthwhile to note that the syntax here is inadequate to represent the orientation of stacking. The specific sequence of the atom indices solely reflects the users' choice on setting the ring index (first atom to start a ring).

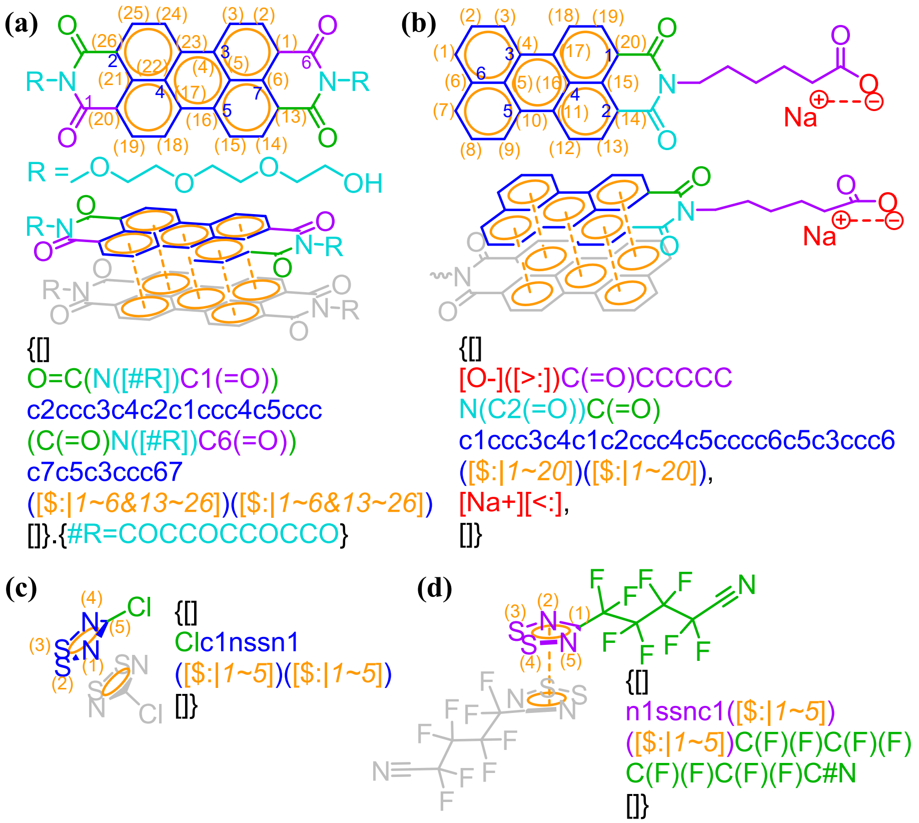

Figure S12. Annotation of π-π stacking in small molecular assemblies. (a) Stacking of tetraethylene glycol (TEG) substituted perylene diimides (PDIs);[35] (b) Stacking of perylene monoimide (PMI) chromophore amphiphile;[36] (c) Stacking of chlorodithiadiazole;[37] (d) Stacking of dithiadiazole with monocycloaddition of octafluoroadiponitrile.[38] Note that in the above figure, different parts of chemical structures are coded with the same colors as their non-covalent BigSMILES strings. The single integers enclosed with round brackets "(i)" are the indices of annotated atoms, while the single integers without brackets "i" are recursive nodes for cyclic structure. The integers enclosed with two layers of square bracket "[[i]j]" are the indices of annotated groups of bonds.

Since stackable planar π systems can interact with each other from either above or below the plane, it is effectively bi-functional and can thus assembled to network structure as illustrated by Fig. S13: The stacking is annotated between monomers of a polymer (panel a, b and c) as well as the polymer side or end groups (panel d and e). Note that in those examples, stacked planar π system are annotated with the identical indexing sequence except for Fig. S13a and c where side groups of aromatic rings, which will not contribute to the stacking, interrupt the sequence of indexing. Therefore, instead of indices being 1~6, the presence of ester and sulfo group interrupt the indexing of the stacked carbon in benzene ring giving rise to 1~3&8~10 in Fig. S13a and 1&6~10 in Fig. S13c, respectively. Since the annotation expression for stacking would be anchored next to the last involved atom character (1-index atom), such that the absolute value of indices for the stacked atoms will not be affected by the location of the annotation expression.

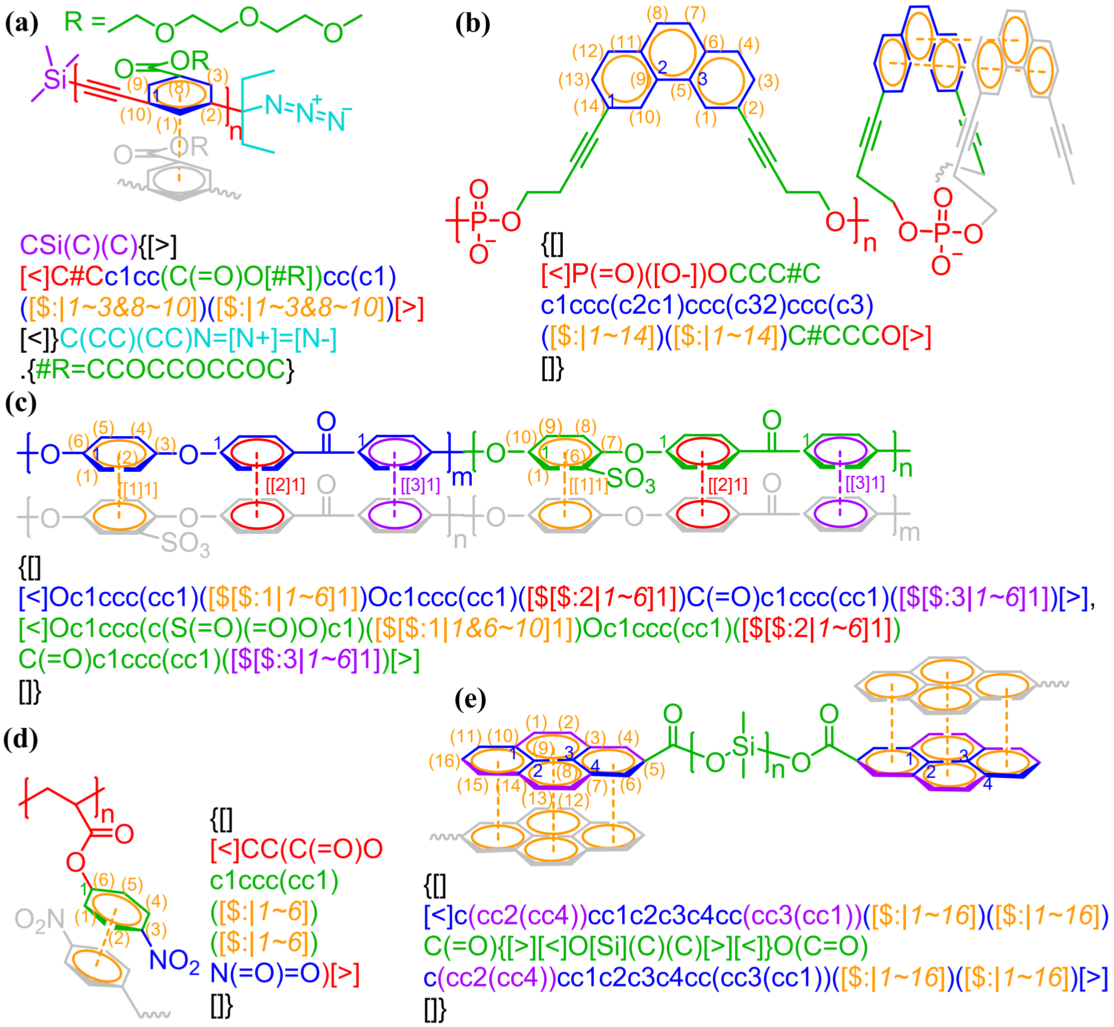

Figure S13. Additional examples showing annotation of π-π stacking with monomers, side and end groups of a polymer. (a) Stacking of meta-substituted phenylacetylene oligomer;[39] (b) stacking of phosphodiester-linked phenanthrene oligomer;[40] (c) stacking of sulfonated poly(ether ether ketone) (SPEEK);[43] (d) π-stacked poly(nitrophenyl methacrylate-co-methacrylic acid);[42] (e) π-stacked network of pyrene end functionalized poly(dimethyl siloxane).[41] Note that in the above figure, different parts of chemical structures are coded with the same colors as their non-covalent BigSMILES strings. The single integers enclosed with round brackets "(i)" are the indices of annotated atoms, while the single integers without brackets "i" are recursive nodes for cyclic structure. The integers enclosed with two layers of square bracket "[[i]j]" are the indices of annotated groups of bonds.

Chain folding & host-guest interactions

The annotation of complicated examples for a folded chain architecture (formed via π-π stacking) are shown in Fig. S14. Once again in Fig. S15 and S16, the potential of the developed annotation formalism is demonstrated by representing different combinations of non-covalent chemistries in building host-guest interactions with complex supramolecular architectures.

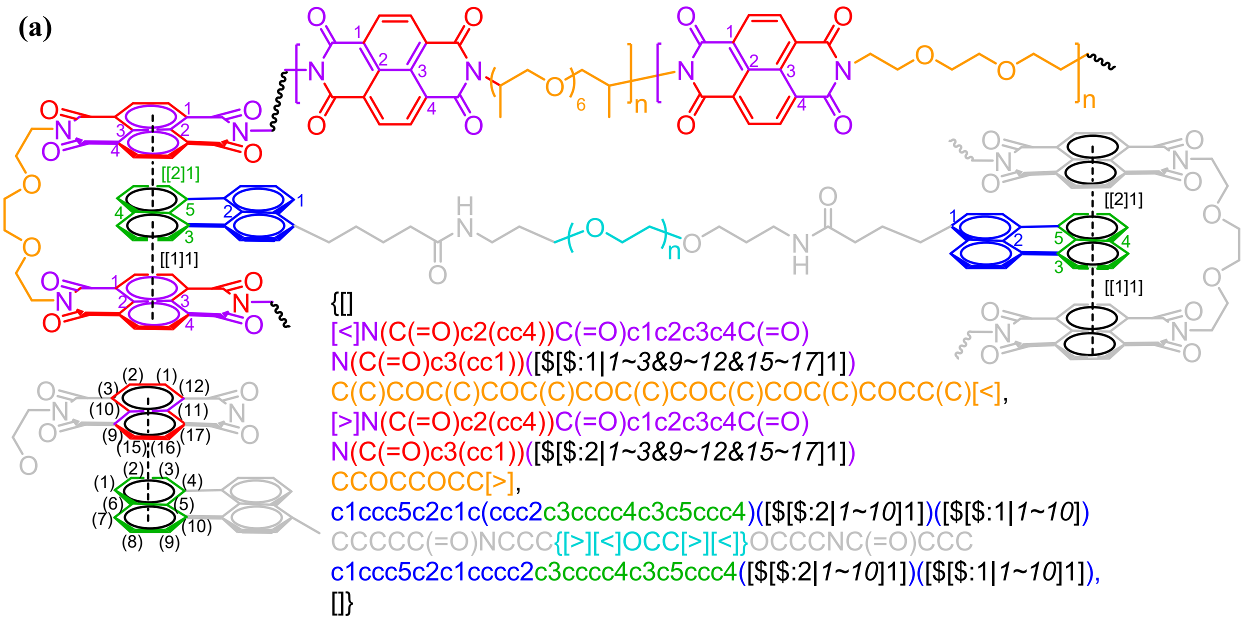

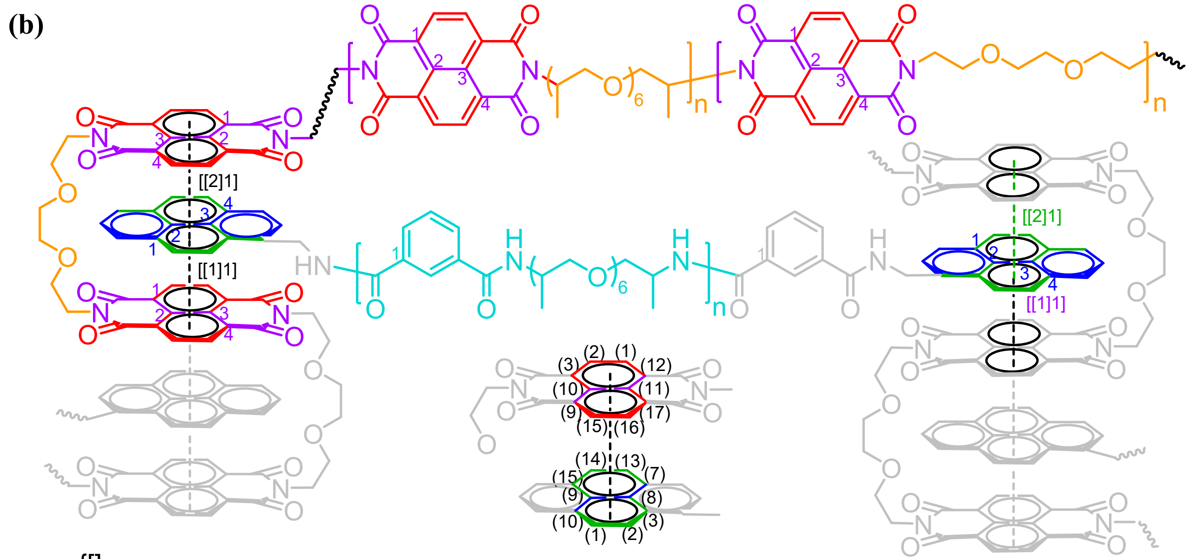

{[]
[<]N(C(=O)c2(cc4))C(=O)c1c2c3c4C(=O)N(C(=O)c3(cc1))([$[$:1|1~3&9~12&15~17]1])
C(C)COC(C)COC(C)COC(C)COC(C)COC(C)COCC(C)[<],
[>]N(C(=O)c2(cc4))C(=O)c1c2c3c4C(=O)N(C(=O)c3(cc1))([$[$:2|1~3&9~12&15~17]1])
CCOCCOCC[>],
c(c2(cc4))ccc1c2c3c4ccc(c3(cc1))([$[$:2|1~3&7~10&13~15]1])([$[$:1|1~3&7~10&13~15]1])
CN{[>][<]C(=O)c1cccc(c1)C(=O)N
C(C)COC(C)COC(C)COC(C)COC(C)COC(C)COCC(C)N[>][<]}C(=O)c1cccc(c1)C(=O)NC
c(c2(cc4))ccc1c2c3c4cccc3(cc1)([$[$:2|1~3&7~10&13~15]1])([$[$:1|1~3&7~10&13~15]1])
[]}

Figure S14. Illustration of π-π stacking patterns and atom indexing in the annotation of folded chain architecture. (a) Chain folding of polydiimide interpolated (triethylenedioxy) naphthalene-diimide with perylene-terminated poly(ethylene glycol);[49] (b) chain-folding triethylenedioxy–diimide motif with terminal pyrenyl residues in polyimides.[44-45] Note that in the above figure, different parts of chemical structures are coded with the same colors as their non-covalent BigSMILES strings. The single integers enclosed with round brackets "(i)" are the indices of annotated atoms, while the single integers without

brackets “i” are recursive nodes for cyclic structure. The integers enclosed with two layers of square bracket “[[i]j]” are the indices of annotated groups of bonds.

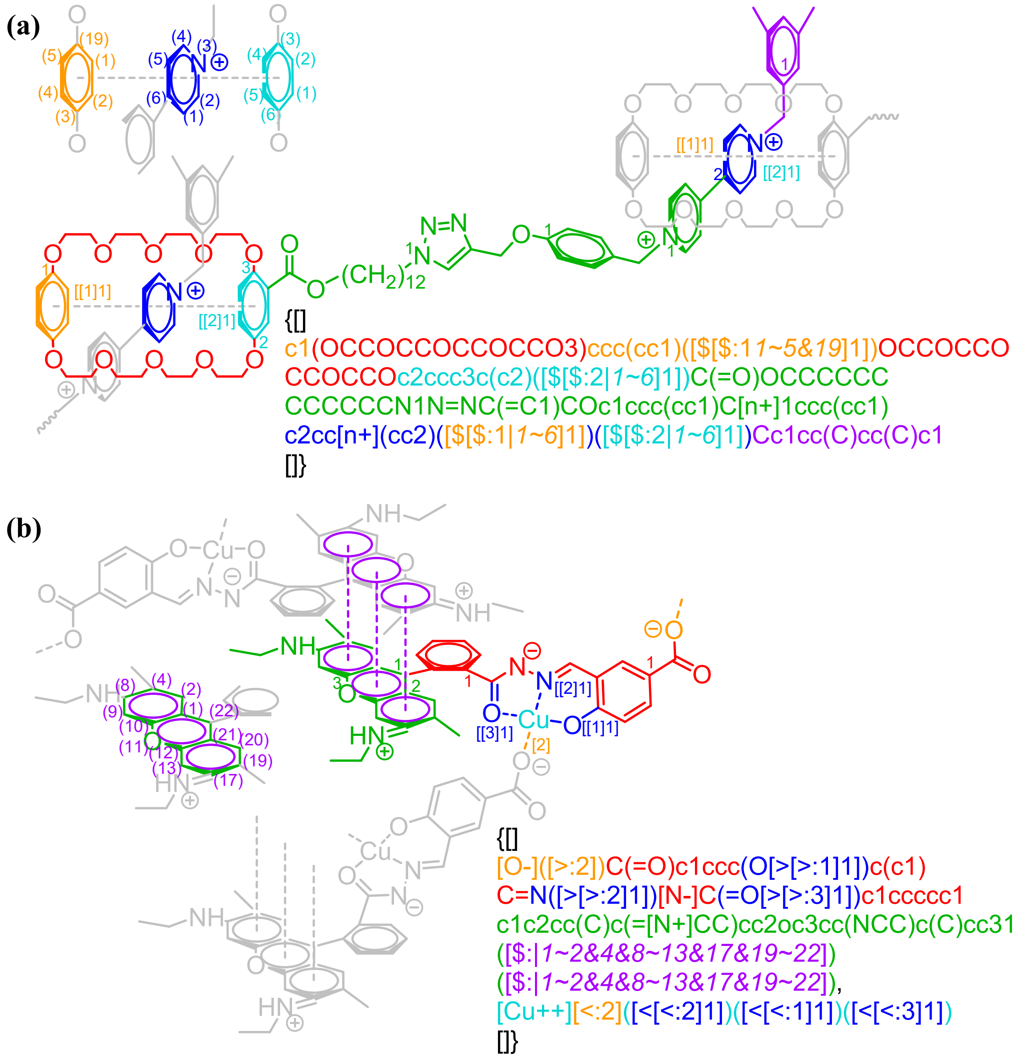

Figure S15. The use of non-covalent BigSMILES syntax to represent the supramolecular assemblies with (a) host-guest architecture of BPP34C10(bis(p-phenylene) crown-10)-paraquat-based analogue[46] and (b) multiple types of non-covalent bonds in Cu^{II} centered salicylaldehyde rhodamine hydrazone derivatives.[47] Note that in the above figure, different parts of chemical structures are coded with the same colors as their non-covalent BigSMILES strings. The single integers enclosed with round brackets “(i)” are the indices of annotated atoms, while the single integers without brackets “i” are recursive nodes for cyclic structure. The integers enclosed with two layers of square bracket “[[i]j]” are the indices of annotated groups of bonds.

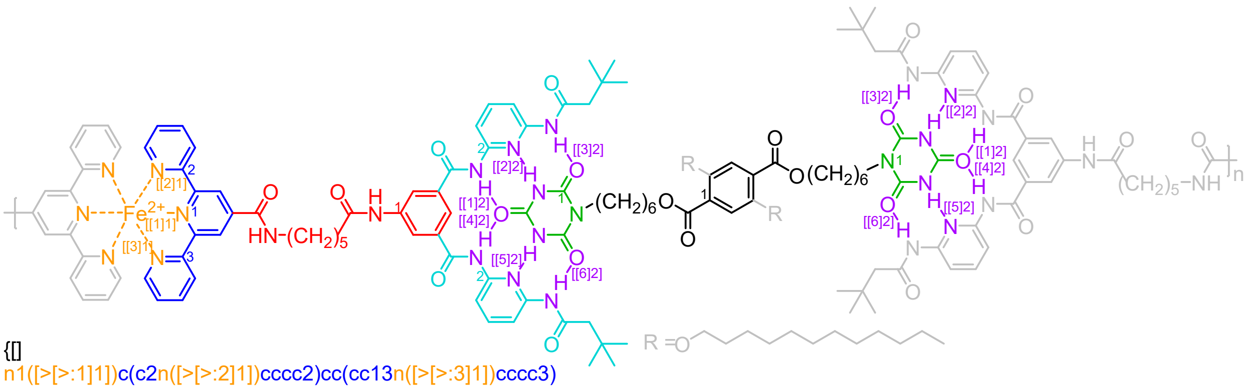


```
{[]
n1([>[>:1]1])c(c2n([>[>:2]1])cccc2)cc(cc13n([>[>:3]1])cccc3)
C(=O)NCCCCCC(=O)Nc1cc(C(=O)N([H][<[<:1]2])c2cccc(n2[<[>:2]2])N([H][<[<:3]2])C(=O)CC(C)(C)C)cc(c1)
C(=O)N([H][<[<:4]2])c2cccc(n2[<[>:5]2])N([H][<[<:6]2])C(=O)CC(C)(C)C,
[Fe++][<[<:1]1]([<[<:2]1])([<[<:3]1])([<[<:3]1])([<[<:2]1])([<[<:1]1]),
O([>[>:3]2])=C1N([H][>[<:2]2])C(=O[>[>:1]2]([>[>:4]2]))N([H][>[<:5]2])C(=O[>[>:6]2])N1
CCCCCCOC(=O)c1cc([#R])c(cc1([#R]))C(=O)OCCCCCC
N1C(=O[>[>:3]2])N([H][>[<:2]2])C(=O[>[>:1]2]([>[>:4]2]))N([H][>[<:5]2])C1(=O[>[>:6]2])
[]}.{#R=OCCCCCCCCCCCC}
```

Figure S16. Another complex example (coupling of terpyridine-carboxylic acid with amine-functionalized Hamilton receptor for coordination of Fe^{II} and multiple H-bonding of cyanuric acid derivatives) showing the success of the annotation in handling host-guest interaction of two different types of non-covalent bonds in forming a supramolecular architecture.[48] Note that in the above figure, different parts of chemical structures are coded with the same colors as their non-covalent BigSMILES strings. The single integers without brackets "i" are recursive nodes for cyclic structure, while the integers enclosed with two layers of square bracket "[[i]j]" are the indices of annotated groups of bonds.

Biopolymers

The inclusion of non-covalent interactions into BigSMILES formalism allows for the meaningful representation of biopolymers. This improvement is achieved by explicitly labelling, for example, the electrostatic interactions and hydrogen bonding between base pairs in nucleic acids or between individual amino acids in a protein sequence. As illustrated by the binding of modified chitosan and heparin shown in Fig. S17a, multiple electrostatic bonding sites and the chirality of carbon atoms in the backbone are properly annotated. In a second example, an artificial spider silk, as illustrated in Fig. S17b, contains a stochastic poly(alanine) and poly(ethylene oxide); the chirality and hydrogen bonding in the poly(alanine) forms the observed beta-sheet formation in this bio-inspired system.[54]

Our group developed a code to accept a sequence of amino acid one-letter symbols that constitute a repeat unit and to output a compact string representation for the protein encoded in BigSMILES with covalent and non-covalent interactions that can be input into polymer databases like PolyDAT. The code is available on GitHub at https://github.com/olsenlabmit/BigSMILES_parser. The repeat unit for each amino acid was first expressed using this code. Non-covalent interactions were then added with the assumption that any hydrogen covalently bound to a nitrogen or oxygen can hydrogen bond with any nitrogen or oxygen with a lone pair. Moreover, electrostatic interactions are encoded for charged amino acids like arginine and histidine. After the user enters a sequence of one-letter amino acid symbols, the code iterates through each one-letter symbol, concatenates the repeat unit of the amino acid, generates a single repeat unit for the user's input sequence, and outputs the non-covalent BigSMILES. Table S1 shows an example of string outputs for three elastin-like polypeptides with different guest residues.

(a)

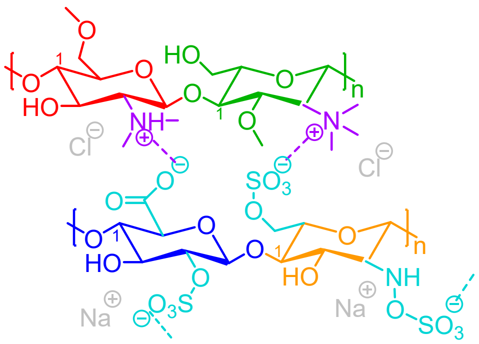

[{[]
[<]O[C@H]1[C@@H](O)[C@H](N(C)C)[C@@H]
(O[C@@H]1(COC))O[C@@H]1[C@H](OC)[C@@H]
([N+]([<:])(C)(C)C)[C@H](O[C@H]1(CO))[>],
[<]O[C@H]1[C@@H](O)[C@H]([N+]([<:])([H])(C)C)[C@@H]
(O[C@@H]1(COC))O[C@@H]1[C@H](OC)[C@@H]
([N+]([<:])(C)(C)C)[C@H](O[C@H]1(CO))[>], [Cl-][>:]
[]}, {[]
[<]O[C@H]1[C@@H](C(=O)O[H])O[C@@H]([C@H]
(OS(=O)(=O)[O-][>:])[C@@H]1(O))O[C@@H]1[C@H]
(COS(=O)(=O)[O-][:>])O[C@H]([C@@H]
(NOS(=O)(=O)[O-][>:])[C@H]1(O))[>],
[<]O[C@H]1[C@@H](C(=O)O[>:])O[C@@H]([C@H]
(OS(=O)(=O)[O-][:>])[C@@H]1(O))O[C@@H]1[C@H]
(COS(=O)(=O)[O-][:>])O[C@H]([C@@H]
(NOS(=O)(=O)[O-][:>])[C@H]1(O))[>], [Na+][<:]
[]}]

(b)

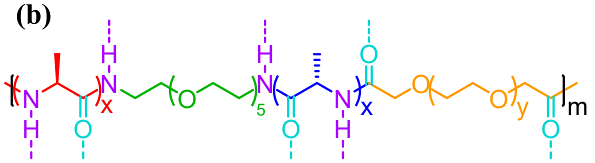

{[]
[$]{[<][>]N([H][<:])[C@H](C)C(=O[>:])[<][>]}
N([H][<:])CCOCCOCCOCCOCCOCCN([H][<:])
{[<][>]C(=O[>:])[C@@H](C)N([H][<:])[<][>]}[$],
[$]C(=O[>:])CO{[>][<]CCO[>][<]}CC(=O[>:])[$]
[]}

Figure S17. Example showing the annotation of the chirality of backbone atoms as well as multiple non-covalent chemistries in biopolymer complexes with N,N,N-trimethyl chitosan and heparin.[50] Note that in the above figure, different parts of chemical structures are coded with the same colors as their non-covalent BigSMILES strings. The single integers without brackets "i" are recursive nodes for cyclic structure, while the integers enclosed with two layers of square bracket "[[i]j]" are the indices of annotated groups of bonds.

Table S1. ELPs encoded in non-covalent BigSMILES $(VPGXG)_n$

Guest residue (X)	BigSMILES
V	{[][<]N([H][<:])[C@@H]([H])(C(C)C)C(=O[>:])N1[C@@H]([H])(CCC1)C(=O[>:])N([H][<:])CC(=O[>:])N([H][<:])[C@@H]([H])(C(C)C)C(=O[>:])N([H][<:])CC(=O[>:])[>][]}
T	{[][<]N([H][<:])[C@@H]([H])(C(C)C)C(=O[>:])N1[C@@H]([H])(CCC1)C(=O[>:])N([H][<:])CC(=O[>:])N([H][<:])[C@@H]([H])([C@H]([H])(O[H][<:])C)C(=O[>:])N([H][<:])CC(=O[>:])[>][]}
A	{[][<]N([H][<:])[C@@H]([H])(C(C)C)C(=O[>:])N1[C@@H]([H])(CCC1)C(=O[>:])N([H][<:])CC(=O[>:])N([H][<:])[C@@H]([H])(C)C(=O[>:])N([H][<:])CC(=O[>:])[>][]}

References

1. Reily, C.; Stewart, T. J.; Renfrow, M. B.; Novak, J., Glycosylation in health and disease. *Nature Reviews Nephrology* **2019,** *15* (6), 346-366.
2. Zhang, Z.; Vaisocherova, H.; Cheng, G.; Yang, W.; Xue, H.; Jiang, S. Y., Nonfouling Behavior of Polycarboxybetaine-Grafted Surfaces: Structural and Environmental Effects. *Biomacromolecules* **2008,** *9* (10), 2686-2692.
3. Chalarca, C. F. S.; Emrick, T., Reactive polymer zwitterions: Sulfonium sulfonates. *Journal of Polymer Science Part a-Polymer Chemistry* **2017,** *55* (1), 83-92.
4. Inoue, Y.; Ishihara, K., Reduction of protein adsorption on well-characterized polymer brush layers with varying chemical structures. *Colloids and Surfaces B: Biointerfaces* **2010,** *81* (1), 350-357.
5. Colak, S.; Tew, G. N., Dual-Functional ROMP-Based Betaines: Effect of Hydrophilicity and Backbone Structure on Nonfouling Properties. *Langmuir* **2012,** *28* (1), 666-675.
6. Zhang, Z.; Chao, T.; Chen, S.; Jiang, S., Superlow Fouling Sulfobetaine and Carboxybetaine Polymers on Glass Slides. *Langmuir* **2006,** *22* (24), 10072-10077.
7. Cao, Z.; Mi, L.; Mendiola, J.; Ella-Menye, J.-R.; Zhang, L.; Xue, H.; Jiang, S., Reversibly Switching the Function of a Surface between Attacking and Defending against Bacteria. *Angewandte Chemie International Edition* **2012,** *51* (11), 2602-2605.
8. Liu, Y.; Duzhko, V. V.; Page, Z. A.; Emrick, T.; Russell, T. P., Conjugated Polymer Zwitterions: Efficient Interlayer Materials in Organic Electronics. *Accounts of Chemical Research* **2016,** *49* (11), 2478-2488.
9. Alswieleh, A. M.; Cheng, N.; Canton, I.; Ustbas, B.; Xue, X.; Ladmiral, V.; Xia, S.; Ducker, R. E.; El Zubir, O.; Cartron, M. L.; Hunter, C. N.; Leggett, G. J.; Armes, S. P., Zwitterionic Poly(amino acid methacrylate) Brushes. *Journal of the American Chemical Society* **2014,** *136* (26), 9404-9413.
10. Colak, S.; Tew, G. N., Amphiphilic Polybetaines: The Effect of Side-Chain Hydrophobicity on Protein Adsorption. *Biomacromolecules* **2012,** *13* (5), 1233-1239.
11. Guan, Z.; Roland, J. T.; Bai, J. Z.; Ma, S. X.; McIntire, T. M.; Nguyen, M., Modular domain structure: a biomimetic strategy for advanced polymeric materials. *J Am Chem Soc* **2004,** *126* (7), 2058-65.
12. Park, T.; Zimmerman, S. C., Formation of a Miscible Supramolecular Polymer Blend through Self-Assembly Mediated by a Quadruply Hydrogen-Bonded Heterocomplex. *Journal of the American Chemical Society* **2006,** *128* (35), 11582-11590.
13. Anthamatten, M., Hydrogen Bonding in Supramolecular Polymer Networks: Glasses, Melts, and Elastomers. In *Supramolecular Polymer Networks and Gels*, Seiffert, S., Ed. Springer International Publishing: Cham, 2015; pp 47-99.
14. Song, G.; Zhang, L.; He, C.; Fang, D.-C.; Whitten, P. G.; Wang, H., Facile Fabrication of Tough Hydrogels Physically Cross-Linked by Strong Cooperative Hydrogen Bonding. *Macromolecules* **2013,** *46* (18), 7423-7435.
15. Yang, Y.; Yang, Z.-Y.; Yi, Y.-P.; Xiang, J.-F.; Chen, C.-F.; Wan, L.-J.; Shuai, Z.-G., Helical Molecular Duplex Strands: Multiple Hydrogen-Bond-Mediated Assembly of Self-Complementary Oligomeric Hydrazide Derivatives. *The Journal of Organic Chemistry* **2007,** *72* (13), 4936-4946.
16. Lewis, C. L.; Stewart, K.; Anthamatten, M., The Influence of Hydrogen Bonding Side-Groups on Viscoelastic Behavior of Linear and Network Polymers. *Macromolecules* **2014,** *47* (2), 729-740.
17. Brunsveld, L.; Vekemans, J. A. J. M.; Hirschberg, J. H. K. K.; Sijbesma, R. P.; Meijer, E. W., Hierarchical Formation of Helical Supramolecular Polymers via Stacking of Hydrogen-Bonded Pairs in Water. *Proceedings of the National Academy of Sciences of the United States of America* **2002,** *99* (8), 4977-4982.
18. Gu, Y.; Alt, E. A.; Wang, H.; Li, X.; Willard, A. P.; Johnson, J. A., Photoswitching topology in polymer networks with metal–organic cages as crosslinks. *Nature* **2018,** *560* (7716), 65-69.

19. Heim, D.; Écija, D.; Seufert, K.; Auwärter, W.; Aurisicchio, C.; Fabbro, C.; Bonifazi, D.; Barth, J. V., Self-Assembly of Flexible One-Dimensional Coordination Polymers on Metal Surfaces. *Journal of the American Chemical Society* **2010,** *132* (19), 6783-6790.
20. Kokado, K., Network polymers derived from the integration of flexible organic polymers and rigid metal–organic frameworks. *Polymer Journal* **2017,** *49* (4), 345-353.
21. Mahmad Rasid, I.; Holten-Andersen, N.; Olsen, B. D., Anomalous Diffusion in Associative Networks of High-Sticker-Density Polymers. *Macromolecules* **2021,** *54* (3), 1354-1365.
22. Mozhdehi, D.; Neal, J. A.; Grindy, S. C.; Cordeau, Y.; Ayala, S.; Holten-Andersen, N.; Guan, Z., Tuning Dynamic Mechanical Response in Metallopolymer Networks through Simultaneous Control of Structural and Temporal Properties of the Networks. *Macromolecules* **2016,** *49* (17), 6310-6321.
23. Nair, K. P.; Breedveld, V.; Weck, M., Multiresponsive Reversible Polymer Networks Based on Hydrogen Bonding and Metal Coordination. *Macromolecules* **2011,** *44* (9), 3346-3357.
24. Paulusse, J. M. J.; Huijbers, J. P. J.; Sijbesma, R. P., Reversible, High Molecular Weight Palladium and Platinum Coordination Polymers Based on Phosphorus Ligands. *Macromolecules* **2005,** *38* (15), 6290-6298.
25. Zhukhovitskiy, A. V.; Zhong, M.; Keeler, E. G.; Michaelis, V. K.; Sun, J. E. P.; Hore, M. J. A.; Pochan, D. J.; Griffin, R. G.; Willard, A. P.; Johnson, J. A., Highly branched and loop-rich gels via formation of metal–organic cages linked by polymers. *Nature Chemistry* **2016,** *8* (1), 33-41.
26. Park, J.; Seo, Y.; Cho, S.; Whang, D.; Kim, K.; Chang, T., Formation of 1,1,12,12-tetramethyl[1.1]silaferrocenophane and poly(ferrocenylsilane) in the reaction of ferrous chloride with the dilithium salt of dicyclopentadienyldimethylsilanes. *Journal of Organometallic Chemistry* **1995,** *489* (1), 23-25.
27. Nugent, H. M.; Rosenblum, M.; Klemarczyk, P., Synthesis of face-to-face metallocene polymers. *Journal of the American Chemical Society* **1993,** *115* (9), 3848-3849.
28. Pittman, C. U.; Ayers, O. E.; McManus, S. P.; Sheats, J. E.; Whitten, C. E., Organometallic Polymers. IX. Polyesters of 1,1'-Bis(chlorocarbonyl)cobalticinium Hexafluorophosphate. *Macromolecules* **1971,** *4* (3), 360-362.
29. Neuse, E. W.; Rosenberg, H., Metallocene Polymers. *Journal of Macromolecular Science, Part C* **1970,** *4* (1), 1-145.
30. Altmann, M.; Enkelmann, V.; Lieser, G.; Bunz, U. H. F., Synthesis of novel polymers containing cyclobutadiene thiophene and alkyne units: polymeric organometallic mesogens**. *Advanced Materials* **1995,** *7* (8), 726-728.
31. Kokil, A.; Shiyanovskaya, I.; Singer, K. D.; Weder, C., High Charge Carrier Mobility in Conjugated Organometallic Polymer Networks. *Journal of the American Chemical Society* **2002,** *124* (34), 9978-9979.
32. Zhang, Y.; Wang, Z.; Kouznetsova, T. B.; Sha, Y.; Xu, E.; Shannahan, L.; Fermen-Coker, M.; Lin, Y.; Tang, C.; Craig, S. L., Distal conformational locks on ferrocene mechanophores guide reaction pathways for increased mechanochemical reactivity. *Nature Chemistry* **2021,** *13* (1), 56-62.
33. Shimura, T.; Ohkubo, A.; Matsuda, N.; Matsuoka, I.; Aramaki, K.; Nishihara, H., Synthesis of Poly(arylene cobaltacyclopentadienylene)s, a New Class of Organometallic π-Conjugated Polymers, by Metallacycling Polymerization and Their Physical Properties. *Chemistry of Materials* **1996,** *8* (6), 1307-1315.
34. Dembek, A. A.; Burch, R. R.; Feiring, A. E., Organometallic modification approach to control of polymer properties: a soluble, liquid crystalline, .pi.-complexed aromatic polyamide. *Journal of the American Chemical Society* **1993,** *115* (5), 2087-2089.
35. Shaller, A. D.; Wang, W.; Gan, H.; Li, A. D. Q., Tunable Molecular Assembly Codes Direct Reaction Pathways. *Angewandte Chemie International Edition* **2008,** *47* (40), 7705-7709.
36. Weingarten, A. S.; Kazantsev, R. V.; Palmer, L. C.; McClendon, M.; Koltonow, A. R.; Samuel, A. P. S.; Kiebala, D. J.; Wasielewski, M. R.; Stupp, S. I., Self-assembling hydrogel scaffolds for photocatalytic hydrogen production. *Nature Chemistry* **2014,** *6* (11), 964-970.

37. Cordes, A. W.; Bryan, C. D.; Davis, W. M.; de Laat, R. H.; Glarum, S. H.; Goddard, J. D.; Haddon, R. C.; Hicks, R. G.; Kennepohl, D. K., Prototypal 1,2,3,5-dithia- and -diselenadiazolyl [HCN2E2].bul. (E = sulfur, selenium): molecular and electronic structures of the radicals and their dimers, by theory and experiment. *Journal of the American Chemical Society* **1993,** *115* (16), 7232-7239.
38. Shuvaev, K. V.; Decken, A.; Grein, F.; Abedin, T. S. M.; Thompson, L. K.; Passmore, J., NC–(CF2)4–CNSSN˙ containing 1,2,3,5-dithiadiazolyl radical dimer exhibiting triplet excited states at low temperature and thermal hysteresis on melting–solidification: structural, spectroscopic, and magnetic characterization. *Dalton Transactions* **2008,** (30), 4029-4037.
39. Nelson, J. C.; Saven, J. G.; Moore, J. S.; Wolynes, P. G., Solvophobically Driven Folding of Nonbiological Oligomers. *Science* **1997,** *277* (5333), 1793-1796.
40. Winiger, C. B.; Li, S.; Kumar, G. R.; Langenegger, S. M.; Häner, R., Long-Distance Electronic Energy Transfer in Light-Harvesting Supramolecular Polymers. *Angewandte Chemie International Edition* **2014,** *53* (49), 13609-13613.
41. Zhuang, W.-R.; Wang, Y.; Cui, P.-F.; Xing, L.; Lee, J.; Kim, D.; Jiang, H.-L.; Oh, Y.-K., Applications of π-π stacking interactions in the design of drug-delivery systems. *Journal of Controlled Release* **2019,** *294*, 311-326.
42. Li, F.; Zhu, Y.; You, B.; Zhao, D.; Ruan, Q.; Zeng, Y.; Ding, C., Smart Hydrogels Co-switched by Hydrogen Bonds and π–π Stacking for Continuously Regulated Controlled-Release System. *Advanced Functional Materials* **2010,** *20* (4), 669-676.
43. Jarumaneeroj, C.; Tashiro, K.; Chirachanchai, S., Shifting from Hydrogen Bond Network to π–π Stacking: A Key Mechanism for Reversible Thermochromic Sulfonated Poly(Ether Ether Ketone). *Macromolecular Rapid Communications* **2014,** *35* (16), 1397-1401.
44. Burattini, S.; Colquhoun, H. M.; Fox, J. D.; Friedmann, D.; Greenland, B. W.; Harris, P. J. F.; Hayes, W.; Mackay, M. E.; Rowan, S. J., A self-repairing, supramolecular polymer system: healability as a consequence of donor–acceptor π–π stacking interactions. *Chemical Communications* **2009,** (44), 6717-6719.
45. Burattini, S.; Greenland, B. W.; Hayes, W.; Mackay, M. E.; Rowan, S. J.; Colquhoun, H. M., A Supramolecular Polymer Based on Tweezer-Type π−π Stacking Interactions: Molecular Design for Healability and Enhanced Toughness. *Chemistry of Materials* **2011,** *23* (1), 6-8.
46. Dong, S.; Yan, X.; Zheng, B.; Chen, J.; Ding, X.; Yu, Y.; Xu, D.; Zhang, M.; Huang, F., A Supramolecular Polymer Blend Containing Two Different Supramolecular Polymers through Self-Sorting Organization of Two Heteroditopic Monomers. *Chemistry – A European Journal* **2012,** *18* (14), 4195-4199.
47. Dong, X.-Y.; Zhang, M.; Pei, R.-B.; Wang, Q.; Wei, D.-H.; Zang, S.-Q.; Fan, Y.-T.; Mak, T. C. W., A Crystalline Copper(II) Coordination Polymer for the Efficient Visible-Light-Driven Generation of Hydrogen. *Angewandte Chemie International Edition* **2016,** *55* (6), 2073-2077.
48. Grimm, F.; Ulm, N.; Gröhn, F.; Düring, J.; Hirsch, A., Self-Assembly of Supramolecular Architectures and Polymers by Orthogonal Metal Complexation and Hydrogen-Bonding Motifs. *Chemistry – A European Journal* **2011,** *17* (34), 9478-9488.
49. Hart, L. R.; Nguyen, N. A.; Harries, J. L.; Mackay, M. E.; Colquhoun, H. M.; Hayes, W., Perylene as an electron-rich moiety in healable, complementary π–π stacked, supramolecular polymer systems. *Polymer* **2015,** *69*, 293-300.
50. Martins, A. F.; Piai, J. F.; Schuquel, I. T. A.; Rubira, A. F.; Muniz, E. C., Polyelectrolyte complexes of chitosan/heparin and N,N,N-trimethyl chitosan/heparin obtained at different pH: I. Preparation, characterization, and controlled release of heparin. *Colloid and Polymer Science* **2011,** *289* (10), 1133-1144.
51. Fulghum, T. M.; Estillore, N. C.; Vo, C.-D.; Armes, S. P.; Advincula, R. C., Stimuli-Responsive Polymer Ultrathin Films with a Binary Architecture: Combined Layer-by-Layer Polyelectrolyte and Surface-Initiated Polymerization Approach. *Macromolecules* **2008,** *41* (2), 429-435.
52. Schmidt, M., *Polyelectrolytes with Defined Molecular Architecture I*. Springer, Berlin, Heidelberg: New York, 2004.

53. Xiao, S.; Ren, B.; Huang, L.; Shen, M.; Zhang, Y.; Zhong, M.; Yang, J.; Zheng, J., Salt-responsive zwitterionic polymer brushes with anti-polyelectrolyte property. *Current Opinion in Chemical Engineering* **2018,** *19*, 86-93.
54. Rathore, O.; Sogah, D. Y., Self-Assembly of β-Sheets into Nanostructures by Poly(alanine) Segments Incorporated in Multiblock Copolymers Inspired by Spider Silk. *Journal of the American Chemical Society* **2001,** *123* (22), 5231-5239.
